# Poly(L-Tyrosine)-Containing Dehydropeptides: Hydrogels vs. Bioadhesives

**DOI:** 10.3390/gels12040305

**Published:** 2026-04-02

**Authors:** Raquel Pereira, Loic Hilliou, Braian E. B. Uribe, José A. Martins, Paula M. T. Ferreira

**Affiliations:** 1 Centre of Chemistry of the University of Minho (CQ-UM), University of Minho, 4710-057 Braga, Portugal; raquelpereira1390@gmail.com; 2 Institute for Polymers and Composites, University of Minho, 4800-058 Guimarães, Portugal; loic@dep.uminho.pt (L.H.); brianes@dep.uminho.pt (B.E.B.U.)

**Keywords:** bioadhesives, tyrosine dehydropeptides, self-assembly, hydrogels, mechanical resilience

## Abstract

Bioadhesive materials capable of operating under aqueous conditions are of considerable interest for biomedical and materials science applications. Peptide-based systems represent an attractive platform for such materials due to their structural tunability, inherent biocompatibility, and ability to form supramolecular networks through noncovalent interactions. In this work, a focused library of tyrosine-containing dehydropeptides was designed and synthesized to investigate how molecular architectures influence self-assembly, hydrogel formation and adhesive properties. The peptides were synthesized using a solution-phase Boc strategy and systematically varied with respect to N-terminal protection and C-terminal functionality. The N-protected dehydropeptides formed supramolecular hydrogels through multiple gelation triggers, including pH reduction and heating–cooling cycles. Rheological characterization confirmed the formation of viscoelastic networks with tunable mechanical properties, with storage moduli reaching tens of kilopascals depending on peptide structure. Scanning electron microscopy revealed dense fibrous nanostructures consistent with supramolecular hydrogel formation. The *N*,*C-*deprotected dehydropeptides displayed reduced gelation propensity but formed cohesive films with measurable adhesive performance toward hydrophilic substrates. Lap-shear tests demonstrated high shear strengths for the hydrophilic films, highlighting their structural robustness under stress. Overall, this study provides insights into the structure–property relationships governing tyrosine-containing dehydropeptide assemblies and demonstrates their potential as minimalistic building blocks for supramolecular adhesive materials.

## 1. Introduction

Bioadhesive materials capable of forming robust interfaces with hydrated surfaces are of increasing interest for applications ranging from wound closure to biomedical coatings. Conventional sutures or staples used in surgical procedures can cause tissue damage and often require invasive application methods. As a result, significant research efforts have focused on the development of soft, biocompatible adhesive materials that operate under physiological conditions. Depending on their composition and intended use, bioadhesives can be subdivided into two main categories: external adhesives, used for skin or superficial wounds, and internal adhesives, formulated to function in wet or internal physiological environments and produce biocompatible and non-toxic degradation products [1,2].

Natural substances such as fibrin, gelatin and albumin provided the first medical adhesives. Subsequent synthetic systems were developed using cyanoacrylates and polymers containing aldehyde functional groups. Despite their excellent bonding strength, these synthetic formulations often release irritant or toxic by-products, which limits their use in internal organs. Consequently, research has shifted toward biopolymer and peptide-based adhesives that combine mechanical strength with biocompatibility and controlled biodegradation [3,4].

The adhesive performance depends on two synergistic parameters: adhesion (interaction with tissue) and cohesion (internal strength of the adhesive). While adhesion relies on the interfacial interactions such as hydrogen bonding and electrostatic attraction, cohesion is maintained by crosslinks and supramolecular interactions within the adhesive matrix. Optimal formulations balance both: excessive cohesion can hinder spreading on tissues, whereas insufficient adhesion leads to premature detachment [5,6].

Supramolecular chemistry is a key driving force in nanotechnology, where peptides stand out as particularly adaptable building blocks. Their molecular features enable them to spontaneously self-assemble into a wide range of nanostructures which can further organize into bulk materials like hydrogels [7,8,9]. These peptide assemblies are held together by noncovalent forces, which confer dynamic and reversible behaviour, allowing self-healing and responsiveness to environmental stimuli. Peptide-based supramolecular materials combine biocompatibility, biodegradability, and modularity, which explains their rapid integration into drug-delivery systems, tissue-engineering scaffolds, biosensors, wound dressings, and regenerative platforms [10,11,12,13,14,15,16].

Self-assembling peptide hydrogels provide a natural scaffold for bioadhesive development due to their hydrated, extracellular matrix (ECM)-mimetic architecture. These systems are composed of more than 99% water yet exhibit semisolid, viscoelastic behaviour, which explains the similarities of their mechanical properties with those of natural tissues [17]. Compared with conventional dressings, these hydrogels maintain a moist healing environment, enable gas exchange, and absorb exudate, all of which accelerate tissue regeneration [13]. Injectable formulations are particularly advantageous as they undergo sol-to-gel transitions in situ, adapting to irregular wound geometries and reducing surgical invasiveness. Commercially available peptide hydrogels are applied in the treatment of chronic, diabetic and burn wounds, and often combine peptides with other biomaterials, such as chitosan, gelatin and hyaluronic acid [18,19,20]. In research, key peptides involved in the development of new peptide-based hydrogels include self-assembling peptides (SAPs), such as RADA16-1 [21], which can rapidly self-assemble into β-sheet nanofibers upon contact with physiological fluids, providing immediate hemeostasis, as well as antimicrobial peptides (AMPs) and L-lysine-containing peptides [22,23,24].

Low-molecular-weight gelators (LMWGs), generally dipeptides or tripeptides, have become the preferred building blocks for minimalistic supramolecular hydrogels [25]. Many different molecular architectures, including *N*,*C*-deprotected peptides, *N*-capped *C*-deprotected peptides, beta-hairpin peptides, amphiphilic and bolaamphiphilic [26,27,28,29,30,31] have been widely explored as effective gelators over the last few decades. All aromatic peptides containing Phe-Phe and Phe-Phe-Phe motifs emerged as exceptionally efficacious gelators [32]. Diaferia et al. comprehensively studied the self-assembly, gelation and properties of hydrogels derived from the all-aromatic peptide motif (Phe-Tyr)_3_ and analogues PEG8-(Phe-Tyr)_3_, (Nal-Tyr)_3_, (Phe-Dopa)_3_, (Nal-Dopa)_3_, (Trp-Y)_3_ and (W-Dopa)_3_, obtained by *N*-functionalization and substitution of the Phe and/or Tyr residues by non-canonical analogues (2-naphthyl alanine- NaI and L-Dopa-*L*-3,4-dihydroxyphenylalanine) and canonical amino acid residues (Trp) [33,34,35,36]. Considering that effective gelation requires a delicate balance between hydrophobic and hydrophilic residues, hydrophobic aromatic residues (Phe, Tyr, Trp) promote π–π stacking and fibril formation, whereas polar or charged residues contribute to solubility and responsive behaviour. Peptides with a low critical gelation concentration (CGC)—often ≤0.5 wt%—are preferred, as minimal mass yields mechanically stable gels [37]. Chemical modification of the *N*-terminus using aromatic groups, such as fluorenylmethoxycarbonyl (Fmoc), carboxybenzyl (Cbz) and naphthaloyl (Naph), is a powerful strategy to enhance gelation by introducing additional π-stacking or hydrophobic interactions [9]. Fmoc *N*-capping is particularly effective in enhancing the self-assembly propensity and gelation ability of low-molecular-weight peptides [35]. The *C-*terminus often remains free and provides a convenient handle for triggering gelation via pH change [38]. However, one major limitation of conventional peptide-based materials is their high susceptibility to enzymatic degradation, which results in short in vivo half-lives and reduced therapeutic or functional stability. To overcome this challenge, researchers often incorporate non-proteinogenic amino acid residues into peptide sequences, including α,β-dehydroamino acids [39]. Research groups, including ours, have reported numerous *N*-capped dehydroamino acid-containing dipeptides and tripeptides as efficient hydrogelators [40,41,42,43,44,45,46].

To improve the adhesive performance, peptide hydrogels are often functionalized with catechols and hydrophobic moieties that magnify interfacial interactions. This outcome mimics aquatic species, especially mussels (*Mytilus* spp.) [47,48], whose remarkable wet adhesion properties have inspired an entire generation of wet-resistant adhesives, due to the presence of foot proteins (MFPs) enriched with L-3,4-dihydroxyphenylalanine (L-DOPA), tyrosine, and lysine residues [49,50,51,52]. As an example, Hauser et al. [53] recently developed a hydrogel resulting from an engineered novel ultrashort peptide sequence incorporating a catechol moiety and lysine residues that demonstrated excellent adhesion to wet surfaces. Primarily designed for coral restoration, the confirmed biocompatibility of this hydrogel also highlights its potential for other clinical applications.

Synthetic analogues employing catechol-bearing monomers or DOPA-modified peptides replicate this functionality. Upon oxidation, catechols convert to quinones capable of reacting with nucleophilic residues (amines, thiols) on tissue surfaces, forming covalent linkages that persist in aqueous conditions. To promote this outcome, new formulations have to employ redox-controlled systems that dynamically modulate catechol oxidation, ensuring strong initial adhesion followed by stable cohesion [54]. Recently, Gazit and co-workers demonstrated that the tripeptide H-Tyr-Tyr-Tyr-OH can self-assemble into a transparent adhesive glass that binds glass slides with lap-shear strengths exceeding 400 kPa [55]. The underlying mechanism involves extensive hydrogen-bond networks between tyrosine hydroxyl groups and water molecules, which facilitate both cohesion and adhesion. The phenolic side chains of tyrosine contribute to wet adhesion, similar to catechols in mussel proteins, but with enhanced oxidative stability. This discovery opens a new paradigm in biomaterial design, where low-molecular-weight peptides can produce solid, transparent, and reconfigurable materials without covalent polymerisation.

**Figure 1:**
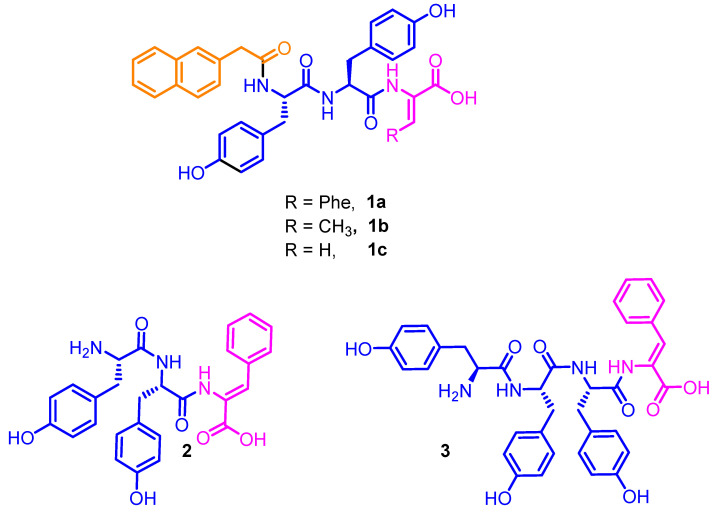
Structure of the tyrosine dehydropeptides synthesized in this work. The poly(Tyr) motif is shown in blue, the dehydroamino acid in pink, and the N-capping group in orange.

In this work, we decided to investigate how tyrosine-containing dehydropeptides behave as building blocks for supramolecular hydrogels and adhesive materials. Tyrosine residues were selected due to their aromatic character and potential to participate in π–π interactions, while the incorporation of dehydroamino acids provides conformational restriction that may influence supramolecular assembly (Figure 1). The dehydropeptide series **1a**–**c** - (Naph-L-Tyr-L-Tyr-∆Xaa; ∆Xaa = ∆Phe, ∆Abu; ∆Ala, respectively) contains a common L-Tyr-L-Tyr dipeptide block N-capped with the bulky aromatic 2-naphthylacetyl group (2-Naph). Dehydropeptide **2** is the N-deprotected (free amine) version of peptide **1a**. Peptide **3** represents the N,C-deprotected tetrapeptide (L-(Tyr)3-∆Phe-OH) version of dehydrotripeptide **2** extended with a Tyr residue. The design of fully deprotected dehydropeptides **2** and **3** as potential adhesives for hydrophilic substrates was inspired by the work of Gazit et al. on adhesive poly(L-Tyr)-OH sequences [55]. The dehydropeptide-based hydrogels were characterized by rheology and their adhesive behaviour was investigated by lap-shear measurements.

## 2. Results and Discussion

### 2.1. Synthesis

**Scheme 1:**
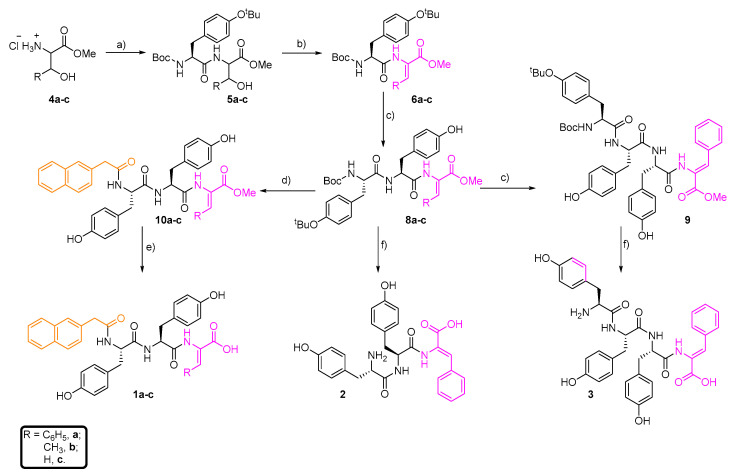
Synthesis of the tyrosine dehydrodipeptides *N-*protected with naphthylacetyl group **1a**–**c**. (a) Boc-L-Tyr(*^t^*Bu)-OH, HBTU, Et_3_N, MeCN; (b) Boc_2_O, DMAP, dry MeCN; TMG; (c) TFA; Boc-L-Tyr(*^t^*Bu)-OH, HBTU, Et_3_N, MeCN; (d) TFA; 2-(naphth-2-yl)acetic acid, HBTU, Et_3_N, MeCN; (e) NaOH; HCl; (f) NaOH; TFA. (The dehydroamino acid residue is shown in pink and the N-capping group is in orange).

Dehydropeptides **1a**–**c** were synthesized using a stepwise *tert*-butoxycarbonyl (Boc) solution-phase protocol previously developed in our research group (Scheme 1) [56]. The Boc-protected dehydropeptide blocks **6** (Scheme 1), with a *O*-tert-butyl tyrosine and the methyl ester of a dehydroamino acid (∆Phe, ∆Abu or ∆Ala), were prepared from the corresponding dipeptides with a β-hydroxyamino acid (β-phenylserine, threonine and serine) (**5**) by treatment with di-*tert*-butyl dicarbonate (Boc_2_O) in the presence of 4-dimethylaminopyridine (DMAP), followed by addition of *N*,*N*,*N′*,*N′*-tetramethylguanidine (TMG) [57]. Protected tripeptides (**8**) were obtained by simultaneous *N*,*O*-deprotection of dipeptides (**6**) with TFA, followed by coupling with Boc-L-Tyr(*^t^*Bu)-OH under standard amidation conditions (HBTU/TEA). Boc deprotection of tripeptides (**8**), followed by reaction with 2-naphthylacetic acid, afforded the 2-Naph-capped methyl ester tripeptides (**10**). The final Naph-capped *C*-deprotected dehydrotripeptides (**1a**–**c**) were obtained by saponification of compounds **10** with NaOH 1M in dioxane. Fully deprotected dehydrotripeptide **2** was obtained from compound **8a** by sequential saponification and TFA deprotection. Dehydrotetrapeptide **3** was synthesized by chain extension of compound **8a** with Boc-L-Tyr(*^t^*Bu)-OH followed by sequential saponification and TFA treatment.

### 2.2. Hydrogel Formation

**Figure 2:**
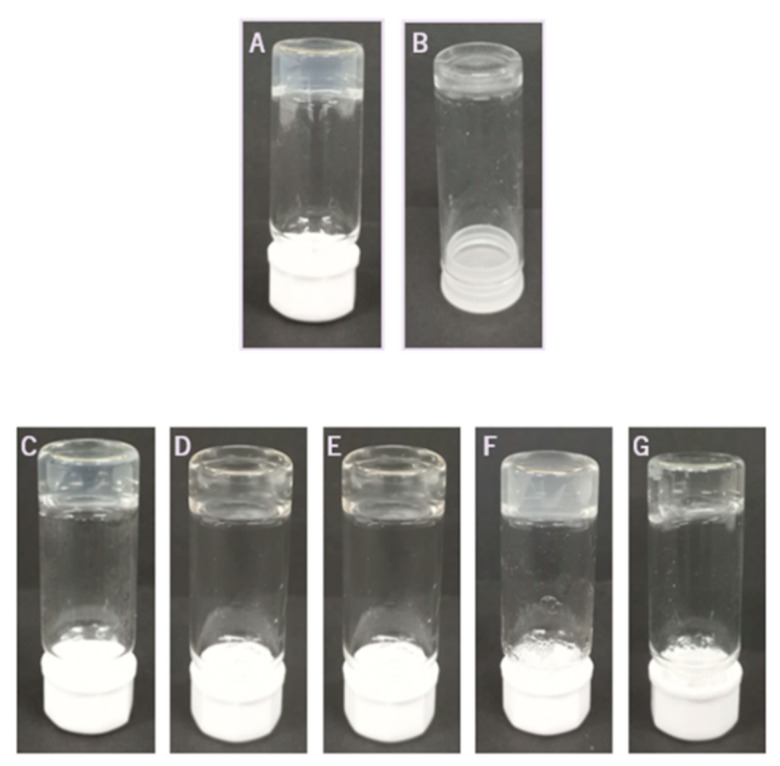
Images of hydrogels obtained using pH and temperature from dehydropeptides **1a**–**c**. (**A**) Hydrogel of **1a** obtained using GdL; (**B**) hydrogel of **1c** obtained using GdL; (**C**) hydrogel of **1a** using solvent switch (DMSO/water); (**D**) **1a** phosphate buffer pH 8, heating–cooling cycle; (**E**) **1a** Na_2_HPO_4_ solution 0.2 M heating–cooling cycle; (**F**) **1b** Na_2_HPO_4_ solution 0.2 M heating–cooling cycle; (**G**) **1c** phosphate buffer pH 6 heating–cooling cycle.

The gelation behaviour of the synthesized peptides was evaluated using several commonly employed triggers for low-molecular-weight gelators, including pH reduction and heating–cooling cycles [58]. Dehydropeptides **1a** and **1c** afforded stable hydrogels (0.5 and 0.6 wt%, respectively) by the GDL-triggered pH drop methodology, whereas peptide **1b** failed to produce gel until 1.0 wt% (Table 1, Figure 2). All three dehydropeptides **1a**–**1c** formed hydrogels by heating–cooling cycles in phosphate buffer or in a 0.2 M Na_2_HPO_4_ solution.

The gelation ability of dehydropeptides **1a**–**c** under different stimuli is consistent with their well-balanced amphiphilic character. Their calculated log P values, within the 2.8–5.5 range, are considered ideal for gelation [25]. The bulky aromatic 2-Naph *N*-capping group is likely to participate in intermolecular π–π stacking interactions that stabilize hydrogels’ fibrous network [9]. The *N*,*C*-deprotected dehydropeptides **2** and **3** failed to originate hydrogels, under all tested conditions, until 1.0 wt%. Presumably, the highly hydrophilic peptides (negative log Pvalues) lack the correct hydrophilicity-hydrophobicity balance that, while ensuring aqueous solubility, promotes pH-triggered hydrophobic collapse, which drives molecular aggregation and the formation of the extended fibrous hydrogels’ structure network. The combined effect of reduced hydrophobicity and lack of an aromatic stacking motif seems to result in insufficient driving force for self-assembly and gelation of compounds **2** and **3**.

### 2.3. SEM Imaging

**Figure 3:**
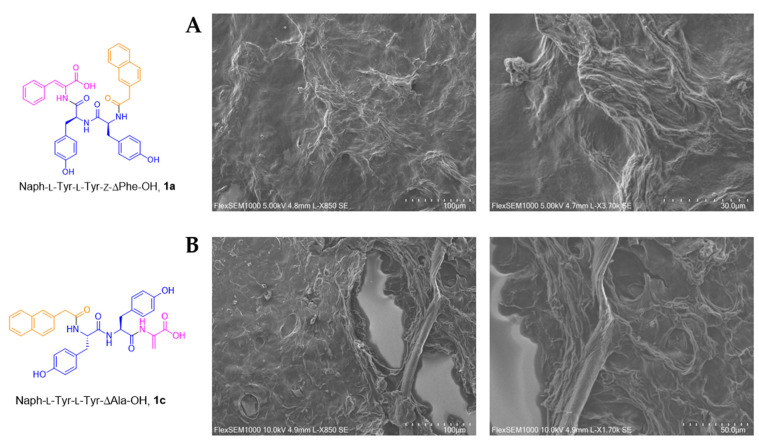
SEM images. (**A**) Hydrogel obtained from compound **1a** (0.5 wt% using GdL), scales 100 µm and 30 µm; (**B**) hydrogel obtained from compound **1c** (0.6 wt% using GdL), scales 100 µm and 50 µm. The poly(Tyr) motif is shown in blue, the dehydroamino acid in pink, and the N-capping group in orange.

The nanostructures of the GdL-triggered hydrogels formed by compounds **1a** (Figure 3A) and **1c** (Figure 3B) were analyzed using scanning electron microscopy (SEM). Compound **1a** self-assembly produces an entangled nanofibrous network with considerable density. This observation is indicative of a mechanically strong hydrogel resulting from an efficient gelation process likely driven by aromatic interactions and π–π stacking involving the dehydrophenylalanine residue. Compound **1c** also forms a dense entangled network, though the fibrils are less defined, suggesting the hydrogel formed is softer/weaker due to a less efficient gelation process in comparison with peptide **1a**. The absence of an aromatic side chain in the dehydroalanine residue can explain the lack of propensity of hydrogelator **1c** in promoting a more robust nanostructure. In general, the fibrils seem not to have uniform thickness for both compounds, with a range between 35 and 58 nm for compound **1a** and 18–30 nm for compound **1c**.

### 2.4. Critical Aggregation Concentrations—Fluorescence Studies

**Figure 4:**
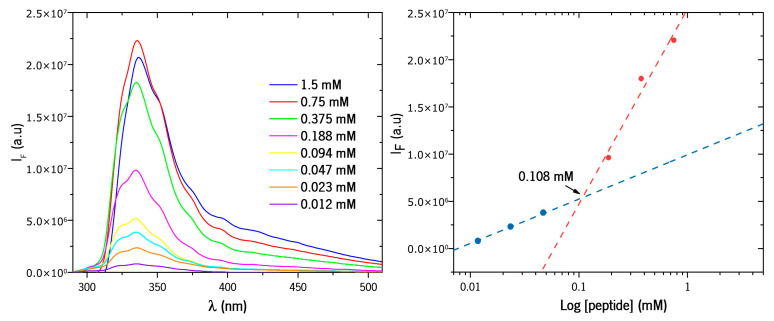
Fluorescence spectra of compounds **1a** in the concentration range between 0.011 mM and 1.5 mM in Na_2_HPO_4_ solution (0.2 M, pH 7.8) (λ_exc_ = 280 nm), and correlation between the fluorescence maximum intensity and the log of concentration.

The critical aggregation concentration (*CAC*) of peptides **1a**–**c** was determined by taking advantage of the intrinsic fluorescence of the 2-Naph capping group [59]. The 2-Naph-capped dehydrotripeptides (**1a**–**c**) exhibited an emission maximum (λ_exc_ = 280 nm) at approximately 335 nm, characteristic of naphthalene monomer emission. At higher concentrations, a broad shoulder near 400 nm, typically associated with aggregates’ emission resulting from π–π stacked naphthalene moieties, often appears (Figure 4 and Figure S1, Supporting Information) [60,61]. In the concentration range studied, the fluorescence spectra of dehydropeptides **1a**–**c** is dominated by naphthalene monomeric emissive species. The CAC values were estimated by plotting the maximum emission intensity vs. log[peptide]; the inflexion point separating the low- and high-concentration regimes was interpreted as the onset of aggregation (Table 2).

**Figure 5:**
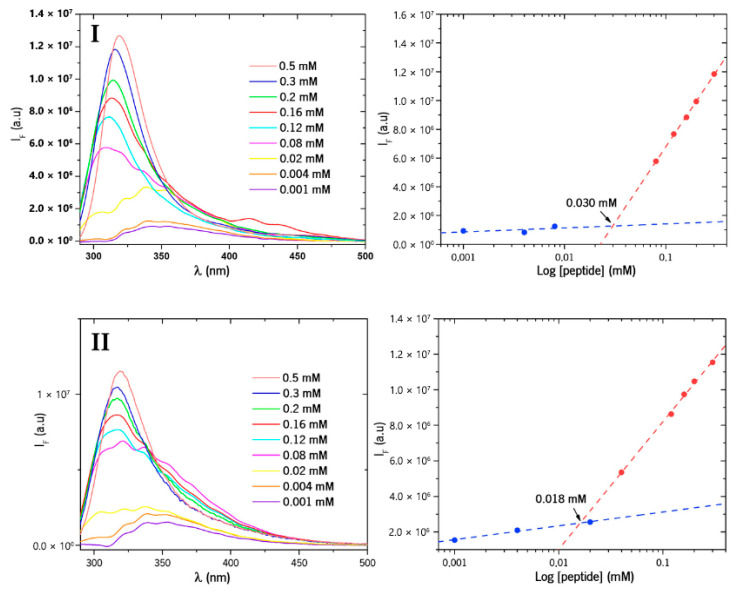
Fluorescence spectra (λ_exc_ = 280 nm) of compounds **2** (**I**) and **3** (**II**) in the concentration range 0.001–0.500 mM in Na_2_HPO_4_ solution (0.2 M, pH 7.8); correlation between the fluorescence maximum intensity and the log of concentration of compounds **2** and **3**.

At low concentrations the fluorescence spectra (λ_exc_ = 280 nm) of dehydropeptides **2** and **3** displayed broad emission bands centred at around 340 nm (Figure 5). The position and shape of the emission band are consistent with the phenolic chromophore of tyrosine: absorption near 230 nm and 280 nm and emission at around 303 nm.

Dehydropeptides **1a** and **3** exhibited the highest and lowest critical aggregation concentration (*CAC* = 0.108 and 0.018 mM, respectively), suggesting that the aggregation propensity of this molecular architecture is primarily driven by direct tyrosine–tyrosine interactions. π–π stacking of the bulky aromatic 2-Naph capping group of compound **1a** seems to play a minor role in directing peptide self-assembly. Presumably, the higher solubility and polarity of the *N*-deprotected compounds promote more efficient hydrogen bonding and π–π interactions between tyrosine residues. Nonetheless, the low *CAC* values of dehydropeptides **2** and **3** do not translate into the formation of extended fibrillar networks and gelation, suggesting weaker long-range π–π stacking between the tyrosine residue in the fully deprotected analogues.

### 2.5. Circular Dichroism

**Figure 6:**
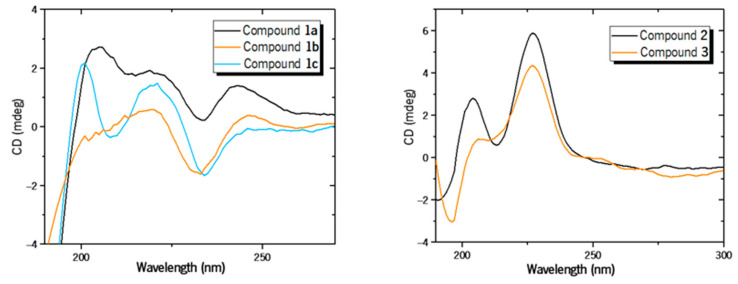
The CD spectra of aqueous solutions of compounds **1a**–**c**, **2** and **3** (0.01 wt%).

The secondary structures of peptides **1a**–**c** was studied by far-UV circular dichroism (CD) under conditions that mimic gelation, although using peptide concentrations (0.1 mg mL^−1^) well bellow the *CGC*. As assemblies in dilute solution are typically less ordered than in the bulk gel phase, the CD spectra are merely indicative of the secondary structure motifs present under pre-gelation conditions rather than exact representations of the gel state (Figure 6).

Hydrogelators **1a**–**c** display overall similar CD spectra: a positive band at around 205 nm and negative bands at around 215 and 234 nm—a pattern consistent with a mixed β-sheet/random-coil signature in short aromatic peptides [62]. The band around ~215 nm supports β-sheet secondary structure assignment, whereas the ~234 nm band reflects chiral packing of aromatic (Tyr) side chains and of the naphthalene capping group. Lacking the band at around 215 nm, the CD spectrum of dehydropeptide **1b** (Naph-*L*-Tyr-*L*-Tyr-*Z*-ΔAbu-OH) indicates that the peptide self-assembly is predominantly mediated by π–π stacking interactions of the aromatic side chains and of the capping group. In contrast, the CD spectrum of dehydropeptide **1c** shows comparatively intense bands both at 215 and 234 nm, highlighting the importance of π–π stacking interactions for supramolecular packing into higher order structures. It is noteworthy that the smallest, least bulky ∆Ala dehydroamino acid seems more prone to induce peptide backbone conformations that support higher order supramolecular packing.

The CD spectra of compound **2** displayed two positive CD bands at 204 nm and 227 nm, alongside a negative band at 213 nm—indicative of β-sheet-like secondary structure. The intense positive signal near 227 nm, attributed to electronic transitions involving phenolic side chains, is characteristic of tyrosine-containing peptides. The CD profile of compound **2** reflects the combined effect of backbone β-sheet organization and aromatic interactions involving tyrosine residues [63]. The CD spectrum of compound **3** also displays the same distinct positive band at 227 nm, characteristic of Tyr, and an initial negative band at 196 nm. Remarkably, the β-sheet signature band at around 215 nm, seen in the CD spectrum of compound **2**, is absent from the CD spectrum of compound **3**, indicating a largely disordered supramolecular packing. Extending the peptide chain with an extra Tyr residue seems to result in lower order supramolecular packing structures.

### 2.6. Rheological Properties

**Figure 7:**
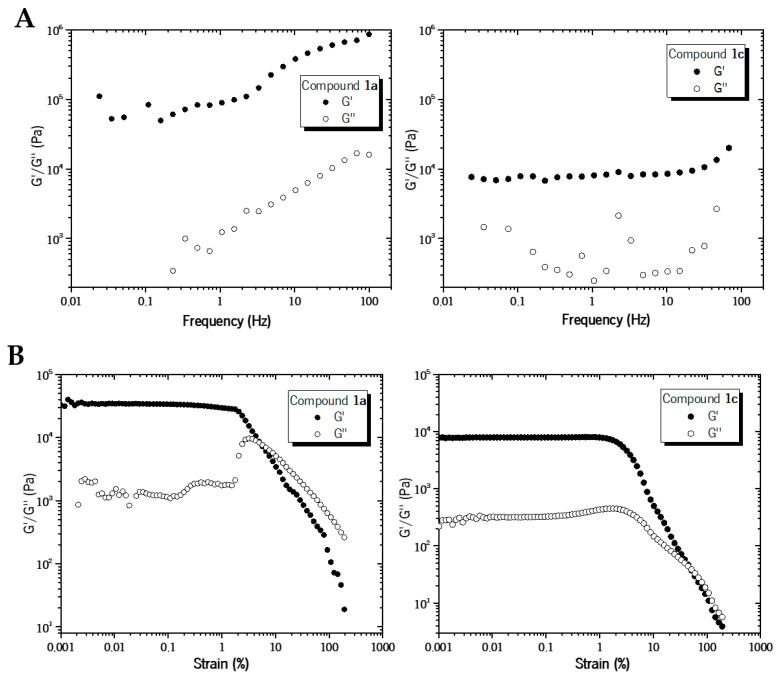
(**A**) Mechanical frequency sweep spectra at constant strain (0.001%) and (**B**) large amplitude oscillatory shear (strain sweep) at a constant frequency (1 Hz) for hydrogels obtained from compounds **1a** and **1c**.

Rheological characterization was performed to elucidate the mechanical behaviour and network architecture of the hydrogels formed by compounds **1a** and **1c** using a pH trigger. Frequency-dependent measurements provided insights into the viscoelastic nature of the gels while strain-amplitude sweeps established the linear viscoelastic region (LVR) and critical strain at failure (Figure 7, Table 3).

**Figure 8:**
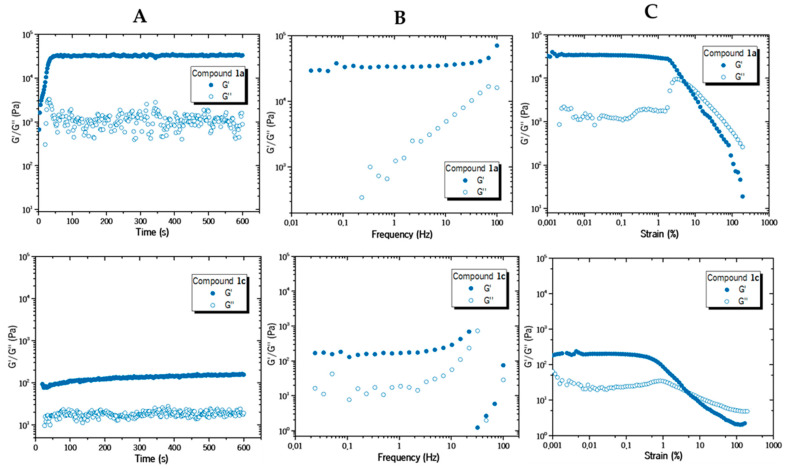
(**A**) Kinetic study of hydrogels’ reformation at constant frequency (f = 1 Hz) and strain (0.01%); (**B**) mechanical frequency sweep at constant strain (0.01%) of the reformed hydrogels; (**C**) large amplitude oscillatory shear (strain sweep) of the reformed hydrogels at a constant frequency (1 Hz).

Both systems displayed a predominantly elastic response (G′ ≫ G″) across the frequency range tested, confirming the formation of a self-supporting fibrillar network characteristic of low-molecular-weight gelators (LMWGs). Hydrogel **1a** exhibited a storage modulus (*G′*~89.6 kPa) around an order of magnitude higher than that displayed by hydrogel **1c** (G′~8.07 kPa), indicating a more rigid and elastic network. Presumably, the dehydrophenylalanine residue promotes stronger π–π stacking and hydrophobic interactions than the dehydroalanine residue, leading to stronger inter-fibrillar contacts that increase the effective crosslink density of the elastic network [64,65]. Hydrogel **1c** showed a much broader linear viscoelastic region than hydrogel **1a** and a much higher critical strain (51.7% compared to 4.97%). Hydrogel **1c** forms a softer yet more ductile network capable of withstanding larger deformations before breakdown. Dense, strongly interacting networks resist small deformations but fail abruptly [66,67]. After mechanical breakdown, both hydrogels **1a** and **1c** exhibited recovery of viscoelastic properties, characteristic of reversible noncovalent crosslinking (Figure 8, Table 4).

Interestingly, hydrogel **1a,** featuring a ∆Phe residue, showed a faster and greater extent of recovery than hydrogel **1c**, containing a ∆Ala residue, indicating faster re-association of π–π stacking and hydrophobic contacts. However, only partial recovery was attained by both hydrogels, suggesting that the reorganization into a less densely crosslinked network is governed by a dynamic array of π–π and hydrogen-bonding interactions [68]. The combination of high stiffness, elastic dominance, and partial self-recovery highlights the potential of these dehydropeptide systems as tuneable supramolecular scaffolds. From an application perspective, the mechanical spectra of **1a** and **1c** span biologically relevant regimes. The high stiffness of **1a** (≈90 kPa) aligns with the range typical of load-bearing soft tissues such as cartilage and tendon, suggesting utility in structural biomaterials or controlled-release platforms. Meanwhile, the softer, more extensible **1c** hydrogel (≈8 kPa) falls within the mechanical window of brain and adipose tissues, making it a suitable candidate for injectable or cell-encapsulating hydrogels where compliance and recoverability are key. The demonstrated ability to recover viscoelastic properties after shear disruption further supports their potential in self-healing or reprocessable supramolecular systems [69,70]. Collectively, the rheological results reveal that the subtle molecular modifications—specifically variations in aromatic content and planarity—profoundly influence the mechanical response, yielding hydrogels that can be rationally tuned along a stiffness–ductility continuum. These findings contribute to the growing understanding of structure–mechanics relationships in peptide-based supramolecular materials.

### 2.7. Adhesive Properties of N,C-Deprotected Dehydropeptide Films

#### 2.7.1. Lap-Shear Screening: Hanging Weight Assay

The adhesive potential of the dehydropeptides’ films was initially evaluated by the hanging weight methodology using overlapping glass slides as a model hydrophilic substrate (Figure S3, Supporting Information). An early screening established the minimum concentrations at which the peptide glues resisted rupture under progressively higher gravitational load (0.5, 1.0, and 1.5 kg) (Table 5, Figure S2, Supporting Information).

Dehydropeptide films of compounds **1a**–**c** required a concentration of 30 mg mL^−1^ to remain intact, whereas films of compounds **2** and **3** succeeded at only 15 mg mL^−1^. When the load was progressively increased, to 1.0 kg and then to 1.5 kg, compounds **2** and **3** maintained cohesion. These results highlight two key points: (i) The self-assembly threshold of **2** and **3** is lower than that of **1a**–**c**, suggesting a more efficient network formation mechanism; and (ii) the peptide films can already sustain a macroscopic load under relatively simple conditions.

#### 2.7.2. Lap-Shear Screening: Controlled Tensile Machine Testing

Lap-shear tests were conducted by controlled tensile machine testing to gain insight into the adhesive performance of dehydropeptide films cast between overlapped glass slides (Figure S3 Supporting Information).

**Figure 9:**
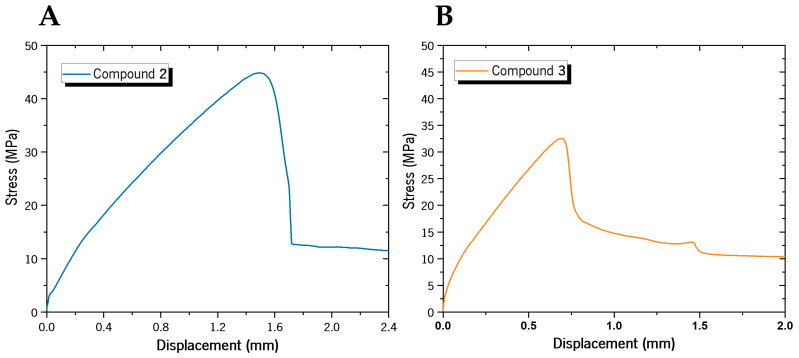
Correlation between the shear stress (MPa) and displacement (mm) during single lap-shear measurements for compounds **2** (**A**) and **3** (**B**) (20 µL of 70 mg mL^−1^ solution, 750 mm^2^ overlap, 5 mm min^−1^).

Using a tensile machine with defined geometry (20 µL of 70 mg mL^−1^ solution, 750 mm^2^ overlap, 5 mm min^−1^) (Figure 9, Figure S4 Supporting Information), compound **3** exhibited a peak nominal shear stress of 32.76 ± 1.93 MPa and failed cohesively. Compound **2**, under identical conditions, reached 44.49 ± 1.07 MPa—a ~36% higher value.

**Figure 10:**
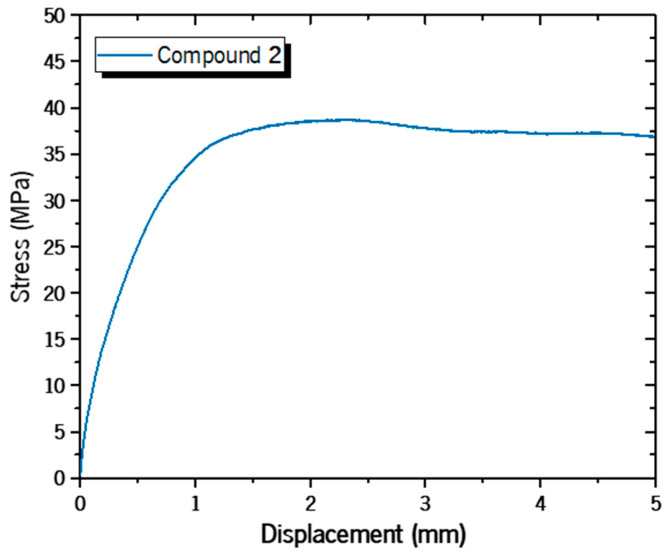
Correlation between the shear stress (MPa) and displacement (mm) during single lap-shear measurements for dehydropeptide **2** (10 µL of 70 mg mL^−1^ solution, 625 mm^2^ overlap, 1 mm min^−1^).

Using 10 µL of dehydropeptide **2** solution (70 mg/mL), a film was formed with a smaller overlapping area between the glass slides (625 mm^2^). The proportionally high overlapping area compared to that produced by 20 µL solution suggests the formation of a thinner film. This film was evaluated with a lower cross speed (1 mm/min). Under this new set of conditions (10 µL of dehydropeptide **2** solution, 70 mg/mL; 625 mm^2^ overlap; cross sped 1 mm/min), the 10 µL dehydropeptide film exhibited a peak shear stress of ~38.44 ± 2.54 MPa, significantly lower than that observed for the 20 µL film (44.49 ± 1.07 MPa), and a continuous displacement superior to 5 mm. The 20 µL film shows displacement of ~0.67 ± 0.02 mm after failure. The 10 µL film shows plastic behaviour within the displacement window (Figure 10). The dehydropeptide film seems to form a more robust adhesive network under these conditions. There are key differences between the peptide films formed by 10 and 20 µL of dehydropeptide solution. In addition to less thickness, faster evaporation kinetics are likely to result in thinner, more uniform films, displaying a more homogeneous fibril network. Moreover, the lower test cross speed allows more time for molecular reorganization of the nanofibrillar network to relieve the applied stress, allowing the adhesive to deform plastically.

Interestingly, films of dehydropeptides **2** and **3** showed significantly higher shear strength than the canonical analogue reported by Gazit et al. (~0.4 MPa, 10 µL of 100 mg mL^−1^ solution, 25 mm^2^ overlap, 1 mm min^−1^) [55]. Moreover, the adhesive behaviour of dehydropeptide **2** could be tuned by adjusting film thickness (solution volume). The thinner (10 µL) peptide film showed a plastic ductile behaviour. The enhanced mechanical performance of the dehydropeptides as adhesives, compared to canonical analogues, suggests that the rigidity of the dehydroamino acids may result in different self-assembly pathways leading to stronger network architectures.

**Figure 11:**
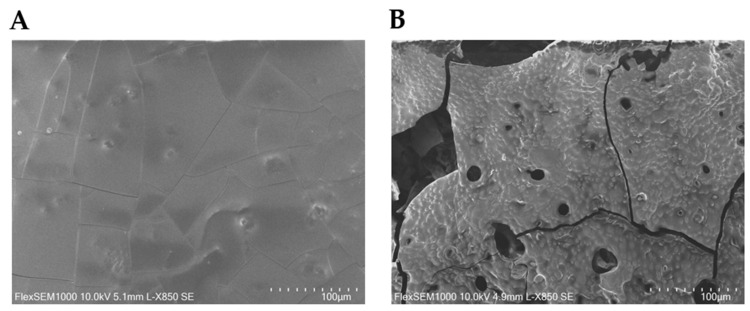
SEM images of the peptide glue of dehydropeptide **2** (**A**) and of dehydropeptide **3** (**B**) (70 mg/mL, scale = 100 µm).

SEM images of the dried adhesive films (Figure 11) reveal clear differences in surface morphology between compounds **2** and **3**, which can be correlated with their mechanical performance in the lap-shear experiments.

Compound **2** (Figure 11A) displays a heterogeneous and moderately textured surface consisting of irregular domains dispersed within a smoother continuous matrix. These domains are sub-micron to micron in size. Such a microstructure would yield a film containing local interfaces and micro voids that can accommodate deformation. This is consistent with the plastic, non-failing behaviour and high lap-shear strength (≈44.5 MPa) observed in compound **2**, particularly in thinner adhesive layers. The microstructural heterogeneity likely enables energy dissipation and progressive strain-hardening, preventing catastrophic fracture. In contrast, compound **3** (Figure 11B) exhibits a densely aggregated and compact microtexture covering the entire surface with little visible open space or smooth background. This morphology suggests that during drying, the peptide assemblies collapse into a uniform, rigid network with limited internal free volume or structural mobility. Such compact aggregation restricts inter-aggregate movement, producing a film that is mechanically strong but brittle. This interpretation aligns with the abrupt cohesive failure observed in the lap-shear test at ≈32.8 MPa. Once microcracks nucleate within this rigid matrix, they likely propagate rapidly, leading to sudden fracture without prior plastic deformation. Taken together, these observations show that the microstructural connectivity of compound **2** correlates with enhanced toughness and energy dissipation, while the compact, rigid architecture of compound **3** leads to higher stiffness but reduced ductility. This structure–property relationship mirrors findings from other peptide-based adhesive systems, where partially fibrillar or heterogeneous morphologies promote superior adhesion and toughness compared with uniformly dense networks [71,72].

The dehydropeptide adhesives reported here achieve lap-shear strengths of 33–45 MPa, substantially exceeding the tensile strengths of previously reported biomimetic adhesives, which typically fall within the 0.01–1 MPa range under hydrated or semi-hydrated conditions (Table S1, Supporting Information) [73]. Traditional bioadhesives like fibrin glue (0.01 MPa) and PEG-based adhesives (0.2 MPa), as well as DOPA-modified peptide/polymer adhesives (0.5 MPa) and mussel-inspired adhesives (7 MPa), display significantly lower tensile strengths compared to the shear strength of compounds **2** and **3**. Despite showing higher tensile strengths, cyanoacrylate adhesives are associated with tissue toxicity, inflammatory responses, and mechanical mismatch with soft biological tissues. The results indicate that the dehydropeptide films perform in the range of structural adhesives commonly used for glass–glass bonding, while retaining the advantages of minimalistic, biocompatible design. The high adhesive strength of the poly(Tyr)-containing dehydropeptides likely arises from a combination of π–π stacking between aromatic residues, enhanced rigidity imparted by the dehydroamino acid residue, and strong interfacial hydrogen bonding between the Tyr hydroxyl groups and the silanol surface of glass.

## 3. Conclusions

Dehydropeptide-based materials emerging from this study collectively demonstrate that minimal, tyrosine-containing sequences can be engineered into robust supramolecular systems that span both hydrogel and high-performance adhesive regimes. The three *N*-naphthylacetyl-protected dehydropeptides **1a**–**c** act as efficient low-molecular-weight hydrogelators, with **1a** in particular forming mechanically reinforced, elastic hydrogels (G’ up to ≈90 kPa) across multiple gelation triggers, underscoring the importance of enhanced aromaticity and optimized hydrophobic balance for dense fibrillar network formation and gel stiffness. In contrast, dehydropeptides **2** and **3** do not gel under the tested conditions but instead yield continuous, mechanically robust films with lap-shear strengths in the 33–45 MPa range, placing them in the performance window of structural glass–glass adhesives while retaining the advantages of peptide-based, potentially biocompatible chemistries (Table S1, Supporting Information). This duality—hydrogelation vs. film formation—highlights a finely tuned interplay between aromatic stacking, backbone rigidity introduced by dehydroamino acid residues, and overall hydrophobicity, which together control both supramolecular architecture and macroscopic mechanical response. The exceptional adhesive performance of compound **2**, coupled with its ductile failure behaviour, suggests that π–π interactions, strong interfacial hydrogen bonding, and film thickness-dependent mechanics can be synergistically coupled to approach or even compete with synthetic structural adhesives. At the same time, variability associated with testing geometry, humidity, and loading protocols emphasizes the need for more standardized, application-relevant mechanical characterization, particularly under hydrated or physiologically mimetic conditions. These results position tyrosine-based dehydropeptides as a versatile platform for designing multifunctional biomaterials in which self-assembly, mechanical robustness, and prospective bioactivity can be co-optimized.

## 4. Materials and Methods

### 4.1. Synthesis

Solvents of analytical grade, purchased from Sigma-Aldrich (Merck), Darmstadt, Germany and Acros Organics (Thermo Fisher Scientific, Waltham, MA, USA), Geel, Belgium were employed and, when necessary, dried using standard procedures. Distilled water was used in all reactions requiring an aqueous medium. Reaction progress was monitored by thin-layer chromatography (TLC) using Merck Kieselgel 60 F254 plates (Merck, Darmstadt, Germany). Visualization was achieved under ultraviolet (UV) light at 240 nm and/or by exposure to iodine vapour. Organic phases were dried over anhydrous magnesium sulfate (Riedel) and anhydrous potassium carbonate (Merck). Chromatographic separations were carried out using silica gel MN Kieselgel 60 M (particle size 230–400 mesh). ^1^H and ^13^C NMR spectra were recorded on a Bruker Avance III 400 spectrometer (Bruker, Billerica, MA, USA) operating at 400.13 MHz and 100.62 MHz, respectively. Signal assignments were supported by DEPT, HSQC, and HMBC techniques. All spectra were acquired at 25 °C using residual solvent signals as internal references. Deuterated dimethyl sulfoxide (DMSO-d_6_) was used as the solvent. Chemical shifts are reported in parts per million (ppm) and coupling constants (J) are given in Hertz (Hz).

#### 4.1.1. Naph-L-Tyr-L-Tyr-Z-ΔPhe-OH (**1a**)

Naph-L-Tyr(*^t^*Bu)-L-Tyr-*Z*-ΔPhe-OMe (**10a**) (0.35 g, 0.52 mmol) was dissolved in 1,4-dioxane (15 mL), and 1 M NaOH (3.0 equiv, 1.56 mmol, 1.56 mL) was added. The reaction was stirred at room temperature and monitored by TLC until complete consumption of the starting material was achieved (≈2 h). Distilled water was added, and the solvent was removed under reduced pressure. The residue was acidified with HCl and left to stand at 4 °C overnight. The resulting precipitate was collected by vacuum filtration to afford Naph-L-Tyr-L-Tyr-*Z*-ΔPhe-OH (**1a**) as a light-yellow solid (0.21 g, 61%). ^1^H NMR (400 MHz, DMSO-*d_6_*, δ): 2.57–2.81 (2H, m, β-C*H*_2_ Tyr), 2.85–2.90 (1H, m, β-C*H*_2_ Tyr), 2.98–3.02 (1H, m, β-C*H*_2_ Tyr), 3.60 (2H, d *J* = 3.6 Hz, C*H*_2_ Naph), 4.43–4.48 (1H, m, α-C*H* Tyr), 4.57–4.62 (1H, m, α-C*H* Tyr), 6.55 (2H, d *J* = 8.4 Hz, Ar*H* Tyr), 6.64 (2H, d *J* = 8.4 Hz, Ar*H* Tyr), 6.97 (2H, d *J* = 8.4 Hz, Ar*H* Tyr), 7.08 (2H, d *J* = 8.4 Hz, Ar*H* Tyr), 7.24 (1H, s, β-C*H* ΔPhe), 7.27–7.83 (12H, m, Ar*H*), 8.20 (1H, d *J* = 8.4 Hz, N*H* Tyr), 8.39 (1H, d *J* = 8.4 Hz, N*H* Tyr), 9.64 (1H, s, N*H* ΔPhe), and 11.97 (1H, br s, O*H*) ppm. ^13^C-NMR (100.6 MHz, DMSO-d_6_, δ): 36.4 and 36.8 (2 × β-CH_2_Tyr), 42.2 (CH_2_ Naph), 54.1 and 54.4 (2 × α-CH Tyr), 114.8 (CH), 114.9 (CH), 125.4 (CH), 126.0 (CH), 127.2 (CH), 127.4 (CH), 127.6 (C), 127.7 (C), 127.9 (CH), 128.5 (CH), 129.9 (CH), 130.1 (CH), 130.2 (CH), 131.4 (β-CH ΔPhe), 131.7 (C), 132.9 (C), 133.6 (C), 133.9 (C), 155.7 (C), 155.8 (C), 166.2 (C=O), 169.8 (C=O), 171.5 (C=O) and 172.1 (C=O) ppm.

#### 4.1.2. Naph-L-Tyr-L-Tyr-Z-ΔAbu-OH (**1b**)

Naph-L-Tyr(*^t^*Bu)-L-Tyr-*Z*-ΔAbu-OMe (**10b**) (0.24 g, 0.39 mmol) was dissolved in 1,4-dioxane (5 mL), and 1 M NaOH (3.0 equiv, 1.18 mmol, 1.18 mL) was added. The reaction mixture was stirred at room temperature and monitored by TLC until complete consumption of the starting material was achieved (≈3.5 h). Distilled water was added, and the solvent was removed under reduced pressure. The residue was acidified with HCl and left at 4 °C overnight. The precipitate was collected by vacuum filtration to afford Naph-L-Tyr(*^t^*Bu)-L-Tyr-*Z*-ΔAbu-OH (**1b**) as a light-yellow solid (0.12 g, 51%). ^1^H NMR (400 MHz, DMSO-*d_6_*, δ): 1.59 (3H, d *J* = 7.2, CH_3_ ΔAbu), 2.74–2.87 (2H, m, β-CH_2_ Tyr), 2.94–2.98 (2H, m, β-CH_2_ Tyr), 3.51 (2H, s, CH_2_ Naph), 4.37–4.39 (1H, m, α-CH of Tyr), 4.46–4.49 (1H, m, α-CH of Tyr), 6.54–6.57 (3H, m, β-C*H* ΔAbu and Ar*H* Tyr), 6.63 (2H, d *J* = 8.4 Hz, Ar*H* Tyr), 6.95 (2H, d *J* = 8.4 Hz, Ar*H* Tyr), 7.06 (2H, d *J* = 8.4 Hz, Ar*H* Tyr), 7.43–7.51 (3H, m, Ar*H* Naph), 7.74–7.87 (4H, m, Ar*H* Naph), 8.11 (1H, d *J* = 8 Hz, N*H* Tyr), 8.20 (1H, d *J* = 8.4 Hz, N*H* Tyr), 9.15 (1H, s, N*H* ΔAbu), and 12.45 (1H, br s, O*H*) ppm. ^13^C-NMR (100.6 MHz, DMSO-d_6_, δ): 13.6 (CH_3_ ΔAbu), 36.0 (CH_2_), 37.7 (CH_2_), 42.2 (CH_2_ Naph), 53.8 (CH), 54.1 (CH), 114. 7 (CH), 114.8 (CH), 125.4 (CH), 125.9 (CH), 127.2 (CH), 127.4 (CH), 130.1 (CH), 130.2 (CH), 131.6 (C), 132.2 (β-C*H* ΔAbu), 132.9 (C), 133.9 (C), 155.7 (C), 155.9 (C),165.4 (C=O), 169.7 (C=O), 169.8 (C=O), and 171.2 (C=O) ppm.

#### 4.1.3. Naph-L-Tyr-L-Tyr-ΔAla-OH (**1c**)

Naph-L-Tyr(*^t^*Bu)-L-Tyr-ΔAla-OMe (**10c**) (0.62 g, 1.05 mmol) was dissolved in 1,4-dioxane (20 mL), and 1.0 M NaOH (3.0 equiv, 3.14 mmol, 3.14 mL) was added. The reaction was stirred at room temperature and monitored by TLC until complete consumption of the starting material was achieved (≈2.5 h). Distilled water was added, and the solvents were removed under reduced pressure. The residue was acidified with HCl and left at 4 °C overnight. The precipitate was collected by vacuum filtration to afford Naph-L-Tyr(*^t^*Bu)-L-Tyr-ΔAla-OH (**1c**) as a light-yellow solid (0.41 g, 67%). ^1^H NMR (400 MHz, DMSO-*d_6_*, δ): 2.57–2.77 (2H, m, β-C*H*_2_ Tyr), 2.84–2.94 (2H, m, β-C*H*_2_ Tyr), 3.51 (2H, s, C*H*_2_ Naph), 4.43–4.47 (1H, m, α-C*H* Tyr), 4.59–4.62 (1H, m, α-C*H* Tyr), 5.72 (1H, s, β-C*H*_2_ ΔAla), 6.29 (1H, s, β-C*H*_2_ ΔAla), 6.55 (2H, d *J* = 6.8 Hz, ArH Tyr), 6.63 (2H, d *J* = 8.4 Hz, ArH Tyr), 6.95 (2H, d *J* = 8.4 Hz, ArH Tyr), 7.04 (2H, d *J* = 8.4 Hz, ArH Tyr), 7.44–7.75 (7H, m, Ar*H* of Naph), 8.29 (1H, d *J* = 8.4 Hz, N*H* Tyr), 8.39 (1H, d *J* = 7.6 Hz, N*H* Tyr), 9.09 (1H, s, N*H* ΔAla), and 13.73 (1H, br s, O*H*) ppm.

^13^C-NMR (100.6 MHz, DMSO-d_6_, δ): 36.0 and 36.8 (CH_2_, β-CH_2_ Tyr), 42.2 (CH_2_ Naph), 54.1 (CH), 55.0 (CH), 108.0 (β-CH_2_ ΔAla), 114.8 (CH), 114.9 (CH), 115.4 (C), 118.7 (C), 121.7 (C), 124.5 (CH), 125.4 (CH), 127.3 (CH), 127.4 (CH), 127.6 (CH), 127.7 (C), 127.8 (C), 130.1 (CH), 131.7 (C),132.6 (C), 132.9 (C), 133.9 (C), 155.7 (C), 155.8 (C), 164.8 (C=O), 169.7 (C=O), 170.6 (C=O), and 171.6 (C=O) ppm.

#### 4.1.4. H-L-Tyr-L-Tyr-Z-ΔPhe-OH·TFA (**2**)

Boc-L-Tyr(*^t^*Bu)-L-Tyr-*Z*-ΔPhe-OH (**11**) (0.27 g, 0.43 mmol) was dissolved in TFA and stirred at room temperature for 1 h. The solvent was removed under reduced pressure. The residue was triturated with Et_2_O, and the solvent was again removed under reduced pressure to afford H-L-Tyr-L-Tyr-*Z*-ΔPhe-OH·TFA (**2**) as a brown solid (0.20 g, 75%). ^1^H NMR (400 MHz, DMSO-*d_6_*, δ): 2.65–2.76 (1H, m, β-C*H*_2_ Tyr), 3.01–3.09 (1H, m, β-C*H*_2_ Tyr), 3.87–3.95 (1H, m, α-C*H* Tyr), 4.68–4.74 (1H, m, α-C*H* Tyr), 6.66 (2H, d *J* = 5.6 Hz, Ar*H* Tyr), 6.68 (2H, d *J* = 6 Hz, Ar*H* Tyr), 7.05 (2H, d *J* = 8.8 Hz, Ar*H* Tyr), 7.12 (2H, d *J* = 8.4 Hz, Ar*H* Tyr), 7.29 (1H, s, β-C*H* Tyr), 7.30–7.58 (5H, m, Ar*H* ΔPhe), 7.95 (3H, d *J* = 4.4 Hz, ^+^N*H*_3_ Tyr), 8.82 (1H, d *J* = 8.4 Hz, N*H* Tyr), and 9.85 (1H, s, N*H* ΔPhe) ppm. ^13^C-NMR (100.6 MHz, DMSO-d_6_, δ): 36.2 and 36.5 (2 × β-CH_2_ Tyr), 53.4 and 54.6 (2 × α-CH Tyr), 115.0 (CH), 115.4 (CH), 128.5 (CH), 129.2 (CH), 129.9 (CH), 130.2 (CH), 130.6 (CH), 131.6 (CH, β-CH ΔPhe), 138.5 (C), 156.0 (C), 156.6 (C),166.1 (C=O), 168.3 (C=O), and 170.8 (C=O) ppm.

#### 4.1.5. H-L-Tyr(^t^Bu)-L-Tyr-L-Tyr-Z-ΔPhe-OH·TFA (**3**)

Boc-L-Tyr(*^t^*Bu)-L-Tyr-L-Tyr-*Z*-ΔPhe-OH (**13**) (0.43 g, 0.53 mmol) was dissolved in trifluoroacetic acid (TFA) and stirred at room temperature for 1 h. The solvent was removed under reduced pressure. The residue was triturated with Et_2_O and concentrated to afford H-L-Tyr(*^t^*Bu)-L-Tyr-L-Tyr-*Z*-ΔPhe-OH·TFA (**3**) as a light-brown solid (0.39 g, 97%). ^1^H NMR (400 MHz, DMSO-*d_6_*, δ): 2.65–2.74 (3H, m, β-C*H*_2_ Tyr), 2.94–3.02 (3H, m, β-C*H*_2_ Tyr), 3.82–3.84 (1H, m, α-C*H* Tyr), 4.50–4.56 (1H, m, α-C*H* Tyr), 4.64–4.70 (1H, m, α-C*H* Tyr), 6.59–6.68 (6H, m, Ar*H* Tyr), 7.02–7.11 (6H, m, Ar*H* Tyr), 7.24 (1H, s, β-C*H* ΔPhe), 7.31–7.56 (5H, m, Ar*H* ΔPhe), 7.88 (3H, d *J* = 3.7 Hz, ^+^N*H*_3_ Tyr), 8.38 (1H, d *J* = 8 Hz, N*H* Tyr), 8.56 (1H, d *J* = 8 Hz, N*H* Tyr), 9.66 (1H, s, N*H* ΔPhe), and 12.18 (1H, br s, O*H*) ppm. ^13^C-NMR (100.6 MHz, DMSO-d_6_, δ): 36.3 (β-CH_2_ Tyr), 36.4 (β-CH_2_ Tyr), 36.9 (β-CH_2_ Tyr), 53.4 (α-CH Tyr), 54.3 (α-CH Tyr), 54.4 (α-CH Tyr), 115.0 (CH), 115.4 (CH), 124.7 (C), 126.7 (C), 127.6 (C), 127.7 (C), 128.5 (CH), 129.2 (CH), 130.0 (CH), 130.1 (CH), 130.6 (CH), 131. 5 (β-CH ΔPhe), 133.6 (C), 155.8 (C), 156.8 (C),166.2 (C=O), 168.0 (C=O), 171.0 (C=O), and 171.1 (C=O) ppm.

### 4.2. Hydrogel Preparation

Hydrogels were prepared using three different methods.

For the pH-triggered (pH-drop) method, the compounds were weighed into sample vials and dissolved in water. Under continuous stirring, 1 M NaOH was added dropwise until the pH reached 10. The resulting mixture was then sonicated, followed by the addition of glucono-δ-lactone (GdL) to induce a gradual decrease in pH. The samples were left undisturbed overnight to allow complete gelation.

For hydrogel formation via heating–cooling cycles, the compounds were weighed into sample vials and dissolved in phosphate buffer solutions at various pH values. The mixtures were first sonicated at room temperature and then heated until complete dissolution was achieved. Subsequently, the solutions were allowed to cool to room temperature, leading to hydrogel formation. In the solvent-switch method, a concentrated solution of the gelator was initially prepared in dimethyl sulfoxide (DMSO). Water was then added slowly in a controlled manner. As the proportion of DMSO decreased, the gelator underwent self-assembly into a three-dimensional network, entrapping the solvent and resulting in hydrogel formation.

### 4.3. Fluorescence Studies

Fluorescence measurements were performed using a Fluoromax-4 spectrofluorometer (Horiba Jobin Yvon, Horiba, France) equipped with double monochromators for both excitation and emission, Glan–Thompson polarizers, and a temperature-controlled cuvette holder. Emission spectra were corrected for the instrument response. Critical aggregation concentrations (CAC) were determined using solutions of peptides **1a**–**c**, **2**, and **3** prepared in 0.2 M Na_2_HPO_4_ buffer (pH 7.8). Fluorescence emission spectra were recorded over the range of 290–540 nm, with an excitation wavelength of 280 nm and a bandwidth of 5 nm.

### 4.4. CD Spectroscopy

Circular dichroism (CD) spectra were recorded under a nitrogen atmosphere using a Jasco J-815 CD spectrometer (Jasco, Tokyo, Japan).The samples were obtained by diluting the hydrogel preparations to appropriate concentrations.

### 4.5. Rheology

Hydrogel viscoelasticity was evaluated using a stress-controlled rotational rheometer (Anton Paar MCR 300, Anton Paar GmbH, Graz, Austria). Gel-forming samples were loaded into a Couette geometry (1 mL volume, 0.5 mm gap) at 25 °C. Prior to measurement, each sample was pre-sheared at a rate of 5 s^−1^ for 1 min to ensure uniformity within the measuring cell. The storage modulus (G′) and loss modulus (G″) were continuously recorded at 1 s intervals throughout the experiment.

### 4.6. Lap-Shear Tests

Shear strength measurements were conducted using a Zwick Roell Z005 tensile testing machine (Zwick Roell GmbH & Co. KG, Ulm, Germany). Thin films were prepared by depositing 10 µL or 20 µL of a 70 mg mL^−1^ peptide solution between two microscope glass slides, forming overlapping contact areas of 625 mm^2^ and 750 mm^2^, respectively. After solvent evaporation, each slide assembly was clamped between the instrument grips. Five replicates of each peptide film were prepared for testing. The shear stress–displacement curves were recorded using a 5 kN load cell, at a crosshead speed of 1 mm min^−1^ (10 µL films) or 5 mm min^−1^ (20 µL films). Figure 9 and Figure 10 presents the plotted data of the three replicates with the best results.

### 4.7. Scanning Electron Microscopy (SEM)

Samples were analyzed using a desktop scanning electron microscope (SEM) (FlexSEM 1000 II, Hitachi High-Technologies Corporation, Tokyo, Japan). Hydrogel and peptide film samples were mounted on aluminum pin stubs using electrically conductive carbon adhesive tape (PELCO Tabs™). Imaging was carried out at an accelerating voltage of 10 kV using a secondary electron (SE) detector.

## Figures and Tables

**Table 1:** Critical gelation concentrations (CGC) and cLogP for tyrosine-based dehydropeptides **1a**–**c**, **2** and **3**.

Hydrogelators	Critical Gelation Concentration (CGC)		Gelation Method	pH	cLogP *
	wt%	mM			
Naph-L-Tyr-L-Tyr-*Z*-ΔPhe-OH, **1a**	0.5	7.6	GdL (0.9 wt%)	4	4.95
	0.4	6.1	DMSO/water	6	
	0.6	9.1	Phosphate buffer/60 °C	8	
	0.4	6.1	Na_2_HPO_4_ (2 M)	7	
Naph-L-Tyr-L-Tyr-*Z*-ΔAbu-OH, **1b**	0.5	8.3	Na_2_HPO_4_ (2 M)	7	4.27
Naph-L-Tyr-L-Tyr-ΔAla-OH, **1c**	0.6	10.3	GdL (1.2 wt%)	4	3.24
	0.6	10.3	Phosphate buffer/60 °C	5	
H-L-Tyr-L-Tyr-*Z*-ΔPhe-OH, **2**	No hydrogel				−0.47
H-L-Tyr-L-Tyr-L-Tyr-*Z*-ΔPhe-OH, **3**	No hydrogel				−0.06

* cLogP calculated LogP values obtained from https//molinspiration.com. (accessed on 15 December 2025).

**Table 2:** Critical aggregation concentration (CAC) of tyrosine-based dehydropeptides **1a**–**c**, **2** and **3** determined by fluorescence spectroscopy.

Dehydropeptide	CAC/mM
Naph-L-Tyr-L-Tyr-Z-ΔPhe-OH, **1a**	0.108
Naph-L-Tyr-L-Tyr-Z-ΔAbu-OH, **1b**	0.065
Naph-L-Tyr-L-Tyr-ΔAla-OH, **1c**	0.082
H-L-Tyr-L-Tyr-Z-ΔPhe-OH, **2**	0.030
H-L-Tyr-L-Tyr-L-Tyr-Z-ΔPhe-OH, **3**	0.018

**Table 3:** Storage (G′) and elastic (G″) moduli and strain for hydrogelators **1a** and **1c**.

Hydrogelator	*G′* (kPa)	*G″* (kPa)	tan *δ* (*G″*/ *G′*)	Strain (%) ^a^
Naph-L-Tyr-L-Tyr-*Z*-ΔPhe-OH, **1a**	89.6	1.24	0.014	4.97
Naph-L-Tyr-L-Tyr-ΔAla-OH, **1c**	8.07	0.247	0.031	51.7

^a^ Critical strain at network breakdown.

**Table 4:** Storage and loss moduli (G′ and G″) strain at breakdown and the recovery percentage from initial G′ value for reformed hydrogels of **1a** and **1c**.

Hydrogelator	*G′* (kPa)	*G″* (kPa)	tan *δ* (*G″*/*G′*)	Recovery (%)	Strain (%) ^a^
Naph-L-Tyr-L-Tyr-*Z*-ΔPhe-OH, **1a**	3.41	1.37	0.40	38.1	4.97
Naph-L-Tyr-L-Tyr-ΔAla-OH, **1c**	0.20	0.022	0.11	1.61	4.29

^a^ Critical strain at network breakdown.

**Table 5:** Calculated *F_R_* (N) and *T* (MPa) for the hanging weight assays with an overlap area of 750 mm^2^.

			Dehydropeptide Film @30 mg/mL					Dehydropeptide Film @15 mg/mL				
Weight (kg)	*F_R_* (N) ^a^	*T* (MPa) ^b^	1a	1b	1c	2	3	1a	1b	1c	2	3
0.50	0.108	6.54 × 10^−3^	√	√	√	√	√	X	X	X	√	√
1.00	0.065	1.31 × 10^−2^	X	X	X	√	√	X	X	X	X	X
1.50	0.082	1.96 × 10^−2^	X	X	X	√	√	X	X	X	X	X

^a^ *F_R_* (N) = *m*. *g*; *m* in the weight mass (kg) and *g* is the gravity acceleration. ^b^ *T* (MPa) = *F_R_*/*A*; A (mm^2^) is the overlapping area of the glass slides.

## Data Availability

The data presented in this study are openly available in the article.

## Supplementary Materials

The following Supporting Information can be downloaded at: https://www.mdpi.com/article/10.3390/gels12040305/s1. Figure S1. Fluorescence spectra of compounds **1b** (A) and **1c** (B) in the concentration range between 0.011 mM and 1.5 mM in Na_2_HPO_4_ solution (0.2 M, pH 7.8) (λ_exc_. = 280 nm) and correlation between the fluorescence maximum intensity and the log of concentration. Figure S2. (A) Schematic representation of the overlapping microscope slides with a downward load; (B) images of the two microscopic slides with peptide solutions and a 0.5 kg downward load. Figure S3. Schematic representation of the single lap-shear tests and results based on the variation in the concentration of the peptide solution. Figure S4. (A) Image of the overlapped glass slides system used in the lap-shear tests; (B) image of the experimental setup for the single lap-shear tests (20 µL of 70 mg mL^−1^ solution, 750 mm^2^ overlap, 5 mm min^−1^). Figure S5. Correlation between the shear stress (MPa) and displacement (mm) during single lap-shear measurements: (A) compound 2 (20 µL, 750 mm^2^ overlap, 5 mm min^−1^); (B) compound 3 (10 µL, 750 mm^2^ overlap, 5 mm min^−1^); (C) compound 2 (10 µL, 625 mm^2^ overlap, 1 mm min^−1^). Table S1. Tensile strength (MPa) of other existing bioadhesives [73].
