# Supplementary material for: Poly(L-Tyrosine)-Containing Dehydropeptides: Hydrogels vs. Bioadhesives
Source: Gels. 2026 Apr 2;12(4):305. doi: 10.3390/gels12040305 (PMC13115828; doi:10.3390/gels12040305)
Supplement: Supplementary file 1 [file gels-12-00305-s001.zip › gels-4227619-supplementary.pdf]

Poly(L-Tyrosine)-Containing Dehydropeptides: Hydrogels vs. Bioadhesives

Raquel Pereira [1], Loic Hilliou [2], Braian E. B. Uribe [2], José A. Martins [1,*] and Paula M. T. Ferreira [1,*]

[1] Centre of Chemistry of the University of Minho (CQ-UM), 4710-057 Braga, Portugal; raquelpereira1390@gmail.com

[2] Institute for Polymers and Composites, University of Minho, 4800-058 Guimarães, Portugal; loic@dep.uminho.pt (L.H.); brianes@dep.uminho.pt (B.E.B.U.)

* Correspondence: jmartins@quimica.uminho.pt (J.A.M.); pmf@quimica.uminho.pt (P.M.T.F.)

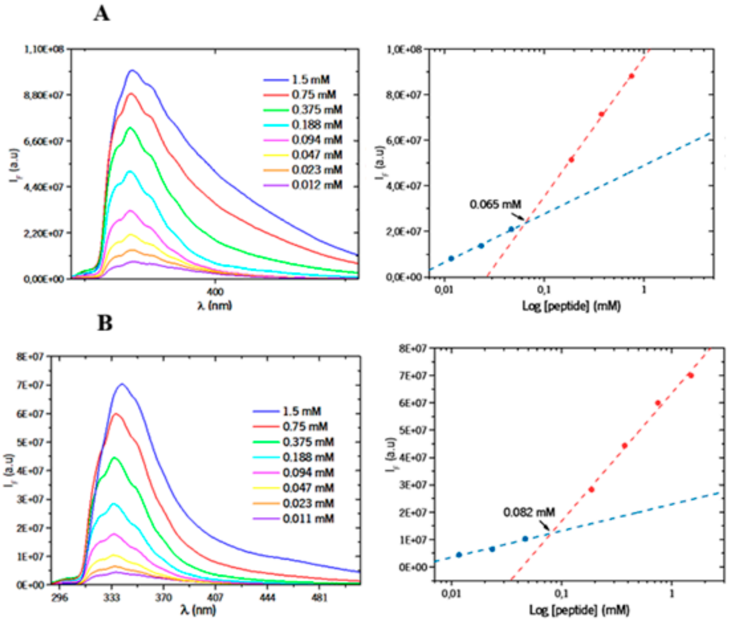

Figure S1. Fluorescence spectra of compounds **1b** (A) and **1c** (B) in the concentration range of 0.011-1.5 mM in Na_2HPO_4 solution (0.2 M, pH 7.8) ($\lambda_{exc.}$ = 280 nm) and correlation between the fluorescence emission maximum intensity and the log of concentration.

Academic Editor: Bruce P. Lee

Received: 13 March 2026
Revised: 27 March 2026
Accepted: 31 March 2026
Published: 2 April 2026

Copyright: © 2026 by the authors. Submitted for possible open access publication under the terms and conditions of the Creative Commons Attribution (CC BY) license.

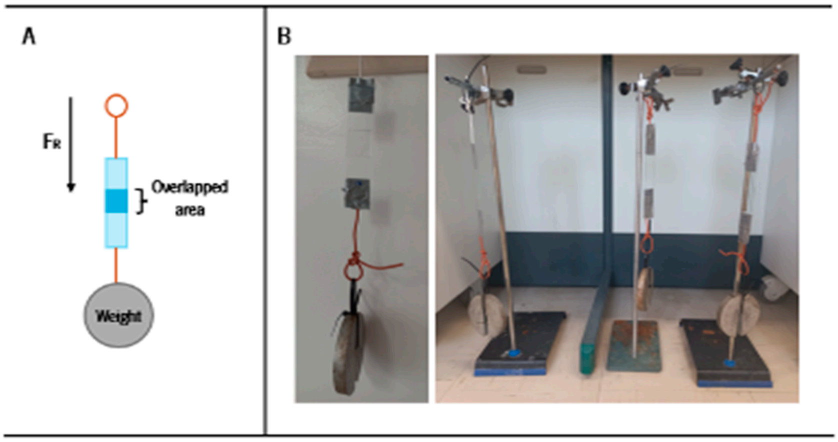

Figure S2. (A) Schematic representation of the overlapping microscope slides with a downward load; **(B)** Images of the two microscopic slides with peptide solutions and a 0.5 kg downward load.

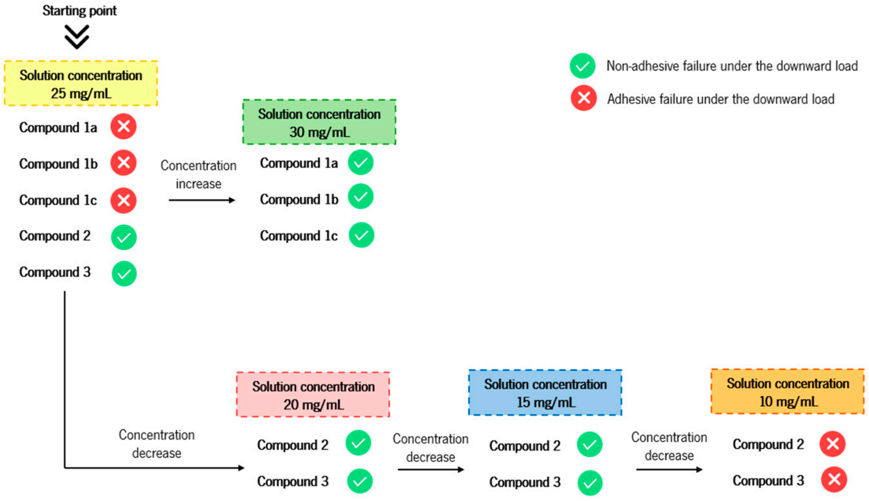

Figure S3. Schematic representation of the single lap-shear tests and results based on the variation of the concentration of the peptide solution.

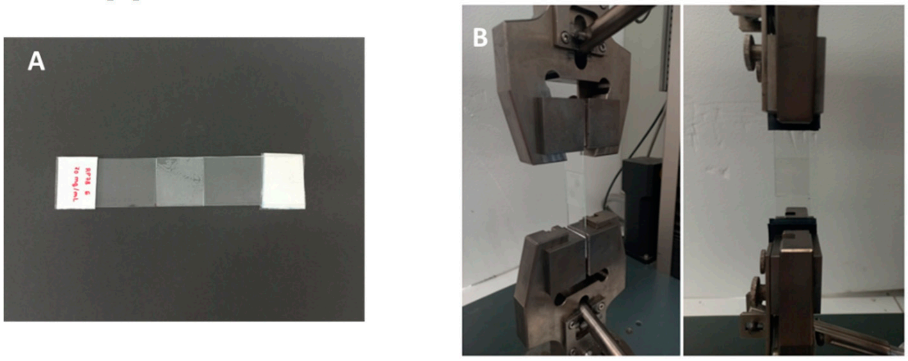

Figure S4. (A) Image of the overlapped glass slides system used in the lap-shear tests; **(B)** Image of the experimental setup for the single lap-shear tests (20 μL of 70 mg mL^{-1} solution, 750 mm^2 overlap, 5 mm min^{-1}).

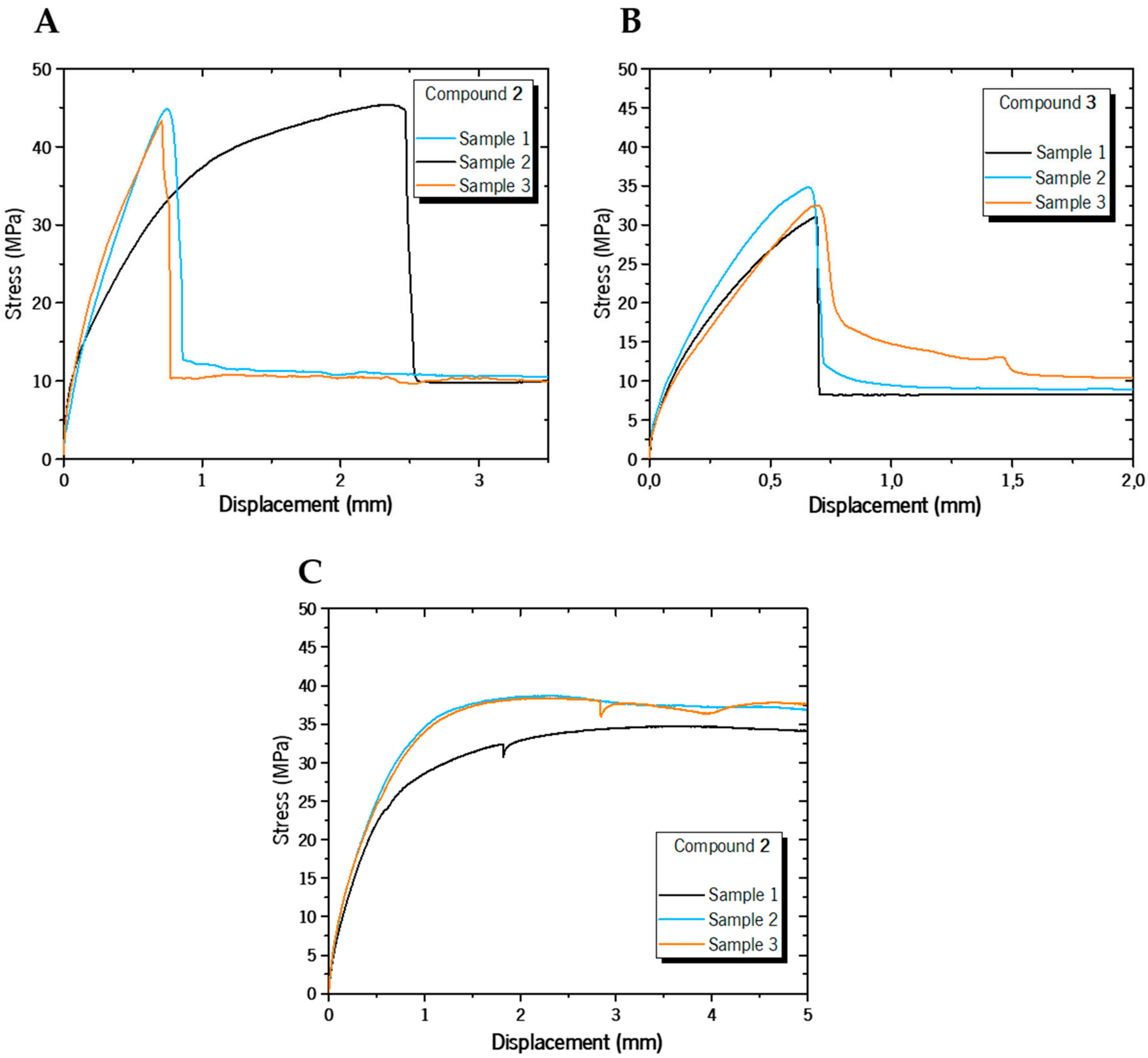

Figure S5. Correlation between the shear stress (MPa) and displacement (mm) during single-lap shear measurements: **(A)** Compound **2** (20 µL of a 70 mg mL^{-1} solution, 750 mm^2 overlap, 5 mm min^{-1}); **(B)** Compound **3** (10 µL of a 70 mg mL^{-1} solution, 750 mm^2 overlap, 5 mm min^{-1}); **(C)** Compound **2** (10 µL of a 70 mg mL^{-1} solution, 625 mm^2 overlap, 1 mm min^{-1}).

Table S1. Tensile strength (MPa) of other existing bioadhesives [73].

Adhesive type	Tensile strength (MPa)
Fibrin glue	0.01-0.02
PEG-based	0.05-0.2
DOPA-modified peptide/polymer	0.01-0.5
Mussel-inspired	< 7
Cyanoacrylate (synthetic)	10-30
Silk fibroin	100-300

Experimental procedures

Compound **4a:**

D,L-3-Phenylserine [H-D,L-Phe(β-OH)-OH] (16.55 mmol, 3.00 g) was dissolved in MeOH (50 mL) at 0 °C. Thionyl chloride (4.0 equiv, 66.2 mmol, 4.80 mL) was added dropwise, and the mixture was refluxed (≈65 °C) overnight. The solvent was removed under reduced pressure. Diethylether (50 mL) was added and evaporated under reduced pressure to afford D,L-Phe(OH)-OMe·HCl (**4a**) (2.82 g, 74%) as a white solid. ^{1}H NMR (400 MHz, DMSO-*d*$_6$, δ): 3.61 (3H, s, OC*H*$_3$), 4.18 (1H, s, β-C*H*), 5.00-5.02 (1H, m, *α*-C*H*), 6.54 (1H, br s, O*H*), 7.30-7.38 (5H, m, Ar*H*), 8.42 (3H, br s, $^+$N*H*$_3$) ppm.

Compound **4b:**

D,L-Threonine (4.50 g, 37.78 mmol, 1.0 equiv) was dissolved in MeOH (50 mL) and cooled to 0 °C. Thionyl chloride (2.0 equiv, 75.56 mmol, 5.48 mL) was added dropwise, and the reaction mixture was stirred under reflux (≈40 °C) overnight. The solvent was removed under reduced pressure, and Et_2O was added and evaporated under reduced pressure to afford H-D,L-Thr-OMe·HCl (**4b**) as a transparent oil (4.47 g, 89%). ^{1}H NMR (400 MHz, DMSO-*d*$_6$,δ): 1.19 (3H, d *J*=6.4 Hz, C*H*$_3$), 3.72 (3H, s, OC*H*$_3$), 3.91 (1H, d *J*=3.2 Hz, *α*-C*H*), 4.09-4.12 (1H, m, β-C*H*), 8.43 (3H, br s, $^+$N*H*$_3$) ppm.

Compound **4c:**

L-Serine (4.50 g, 42.82 mmol, 1.0 equiv) was dissolved in MeOH (50 mL) and cooled to 0 °C. Thionyl chloride (2.0 equiv, 85.64 mmol, 6.21 mL) was added dropwise, and the mixture was refluxed (≈65 °C) overnight. The solvent was removed under reduced pressure, then Et_2O was added and evaporated to afford H-L-Ser-OMe·HCl (**4c**) as a white solid (4.46 g, 67%). ^{1}H NMR (400 MHz, DMSO-*d*$_6$, δ): 3.72 (3H, s, OC*H*$_3$), 3.80-3.81 (2H, m, β-C*H*$_2$), 3.98-4.06 (1H, m, *α*-C*H*), 8.58 (3H, br s, $^+$N*H*$_3$) ppm.

Compound **5a:**

H-D,L-Phe(β-OH)-OMe (**4a**) (1.0 equiv, 5.12 mmol, 1.00 g) was dissolved in MeCN (20 mL) and cooled to 0 °C. HBTU (1.2 equiv, 6.14 mmol, 2.33 g) was added, and after 2 min Boc-L-Tyr(^{t}Bu)-OH (1.0 equiv, 5.12 mmol, 1.73 g) and triethylamine (3.0 equiv, 15.36 mmol, 2.14 mL) were added sequentially. The reaction mixture was stirred at room temperature overnight. The solvent was removed under reduced pressure, and the residue was partitioned between EtOAc (50 mL) and 1 M $KHSO_4$ (50 mL). The organic layer was washed with 1 M $KHSO_4$ (2 × 50 mL), 1 M $NaHCO_3$ (2 × 50 mL), and brine (50 mL), then dried over $MgSO_4$. Filtration and solvent removal afforded a diastereomeric mixture of Boc-L-Tyr(^{t}Bu)-D,L-Phe(β-OH)-OMe (**5a**) as a yellow oil (2.34 g, 89%). ^{1}H NMR (400 MHz, DMSO-*d*$_6$, δ): 1.27 (9H, s, 3 x C*H*$_3$ ^{t}Bu), 1.30 (9H, s, 3 x C*H*$_3$ Boc), 2.62-2.73 (2H, m, β-C*H*$_2$ Tyr), 3.66 (3H, s, OC*H*$_3$), 4.11-4.19 (1H, m, β-C*H* Phe(β-OH)), 4.52-4.60 (1H, m, *α*-C*H* Phe(β-OH)), 5.11-5.17 (1H, m, *α*-C*H* Tyr), 6.70-7.41 (18H, m, Ar*H*), 7.98-8.00 (1H, d *J*=8.8 Hz, N*H*), 8.18-8.20 (1H, d *J*=9.2 Hz, N*H*) ppm.

Compound **5b:**

H-D,L-Thr-OMe.HCl (**4b**) (1.0 equiv, 7.51 mmol, 1 g) was dissolved in MeCN (20 mL) and cooled to 0 °C. HBTU (1.2 equiv, 9.01 mmol, 3.41 g) was added, and after 2 min Boc-L-Tyr(tBu)-OH (1.0 equiv, 7.51 mmol, 2.53 g) and triethylamine (3.0 equiv, 22.53 mmol, 3.10 mL) were added sequentially. The reaction mixture was stirred at room temperature overnight. The solvent was removed under reduced pressure, and the residue was partitioned between EtOAc (50 mL) and 1 M $KHSO_4$ (50 mL). The organic layer was washed with 1 M $KHSO_4$ (2 × 50 mL), 1 M $NaHCO_3$ (2 × 50 mL), and brine (50 mL), then dried over $MgSO_4$. Filtration and solvent removal under reduced pressure afforded a diastereomeric mixture of Boc-L-Tyr(^{t}Bu)-D,L-Thr-OMe (**5b**) as a yellow oil (2.14 g, 63 %). ^{1}H NMR (400 MHz, DMSO-d_6, δ): 1.06 (3H, d *J*=6.4 Hz, C*H*$_3$ Thr), 1.28 (9H, s, 3 x C*H*$_3$ ^{t}Bu), 1.30 (9H, s, 3 x C*H*$_3$ Boc), 2.65-2.71 (1H, m, β-C*H*$_2$ Tyr), 2.92-2.98 (1H, m, β-C*H*$_2$ Tyr), 3.61 (3H, s, OC*H*$_3$), 4.00-4.29 (2H, m, *α*-C*H* Thr and *α*-C*H* Tyr), 4.68-4.72 (1H, m, β-C*H* Thr), 6.85 (2H, d *J*=8 Hz, Ar*H* Tyr), 6.98-7.02 (1H, m, N*H* of Tyr), 7.17 (2H, d *J*=8.4 Hz, Ar*H* Tyr), 7.83-7.89 (1H, m, N*H* Thr) ppm. ^{13}C-NMR (100.6 MHz, DMSO-d$_6$, δ): 14.1 (CH_3 Thr), 28.1 and 28.5 (OC(CH_3)$_3$ ^{t}Bu and Boc), 36.6 and 36.9 (2 x β-CH_2 Tyr), 51.9 (OCH_3), 55.7 (CH), 55.9 (CH), 77.6 and 78.1 (O*C*(CH_3)$_3$ ^{t}Bu and Boc), 123.3 (CH), 123.5 (CH), 132.7 (CH), 132.8 (CH) 153.4 (C), 153.6 (C), 155.2 (C), 155.3 (C), 170.4 (C=O), 171.0 (C=O), 172.3 (C=O) ppm.

Compound **5c:**

The methyl ester of serine (**4c**) (1.00 g, 8.39 mmol, 1.0 equiv) was dissolved in MeCN (20 mL) and cooled to 0 °C. HBTU (1.2 equiv, 10.07 mmol, 3.82 g) was added, and after 2 min, Boc-L-Tyr(^{t}Bu)-OH (1.0 equiv, 8.39 mmol, 2.83 g) and triethylamine (3.0 equiv, 25.17 mmol, 3.51 mL) were added sequentially. The reaction mixture was stirred at room temperature overnight. The solvent was removed under reduced pressure, and the residue was partitioned between EtOAc (50 mL) and 1 M $KHSO_4$ (50 mL). The organic layer was washed with 1 M $KHSO_4$ (2 × 50 mL), 1 M $NaHCO_3$ (2 × 50 mL), and brine (50 mL), then dried over $MgSO_4$. Filtration and solvent removal under reduced pressure afforded a diastereomeric mixture of Boc-L-Tyr(^{t}Bu)-L-Ser-OMe (**5c**) as a yellow oil (2.17 g, 61%). ^{1}H NMR (400 MHz, DMSO-d_6, δ): 1.27 (9H, s, 3 x C*H*$_3$), 1.28 (9H, s, 3 x C*H*$_3$), 2.67-2.78 (1H, m, β-CH_2 Tyr), 2.91-2.98 (1H, m, β-CH_2 Tyr), 3.65 (3H, s, OC*H*$_3$), 4.18-4.26 (2H, m, β-CH_2 Ala), 4.35-4.36 (1H, m, *α*-C*H* Ser), 4.63-4.65 (1H, m, *α*-C*H* Tyr), 6.83-7.23 (8H, m, Ar*H*), 8.46 (1H, d *J*=7.6, N*H*) ppm. ^{13}C-NMR (100.6 MHz, DMSO-d$_6$, δ): 28.0 and 28.5 (OC(CH_3)$_3$ ^{t}Bu and Boc), 35.6 (CH_2), 36.8 (CH_2), 52.3 (OCH_3), 54.9 (CH), 55.4 (CH), 64.9 (CH_2, β-CH_2 Ser), 77.5 and 77.6 (O*C*(CH_3)$_3$ Boc and ^{t}Bu), 123.3 (CH), 123.4 (CH), 153.3 (C), 155.1 (C), 169.6 (C=O), 171.7 (C=O), 172.1 (C=O) ppm.

Compound **6a:**

DMAP (0.11 equiv, 0.55 mmol, 0.07 g) and di-*tert*-butyl dicarbonate (Boc_2O, 1.1 equiv, 5.01 mmol, 1.07 g) were added to a solution of Boc-L-Tyr(^{t}Bu)-D,L-Phe(β-OH)-OMe (**5a**) (1.0 equiv, 4.55 mmol, 2.34 g) in dry MeCN (10 mL). The reaction mixture was stirred at room temperature overnight, and progress was monitored by ^{1}H NMR until complete consumption of the starting material. *N,N,N′,N′*-Tetramethylguanidine (2% v/v, 0.20 mL) was then added, and stirring was continued at room temperature, again monitoring the reaction by ^{1}H NMR until the intermediate was fully consumed. The solvent was removed under reduced pressure, and the residue was partitioned between

EtOAc (50 mL) and 1 M $KHSO_4$ (50 mL). The organic phase was washed with 1 M $KHSO_4$ (2 × 50 mL), 1 M $NaHCO_3$ (2 × 50 mL), and brine (50 mL), then dried over $MgSO_4$. Filtration and solvent removal under reduced pressure afforded Boc-L-Tyr(^{t}Bu)-Z-ΔPhe-OMe (**6a**) as a yellow oil (1.41 g, 63%). ^{1}H NMR (400 MHz, DMSO-d_6, δ): 1.29 (9H, s, 3 x CH_3 ^{t}Bu), 1.30 (9H, s, 3 x CH_3 Boc), 2.69-2.75 (1H, m, β-CH_2 Tyr), 2.97-3.01 (1H, m, β-CH_2 Tyr), 3.66 (3H, s, OCH_3), 4.12-4.16 (1H, m, α-C*H* Tyr), 6.83-6.87 (3H, m, Ar*H* Tyr), 7.01-7.11 (1H, m, N*H* Tyr), 7.20-7.24 (2H, Ar*H* Tyr and β-C*H* ΔPhe), 7.36-7.68 (5H, m, Ar*H* ΔPhe), 9.76 (1H, s, N*H* ΔPhe) ppm.

Compound **6b:**

DMAP (0.22 equiv, 0.57 mmol, 0.07 g) and di-*tert*-butyl dicarbonate (Boc_2O, 1.5 equiv, 3.86 mmol, 0.83 g) were added to a solution of Boc-L-Tyr(^{t}Bu)-D,L-Thr-OMe (**5b**) (1.0 equiv, 2.57 mmol, 1.16 g) in dry MeCN (8 mL). The mixture was stirred at room temperature overnight, and progress was monitored by ^{1}H NMR until complete consumption of the starting material. *N,N,N′,N′*-tetramethylguanidine (TMG, 2% v/v, 0.16 mL) was then added, and stirring at room temperature was continued, monitoring by ^{1}H NMR until the intermediate was fully consumed. The solvent was removed under reduced pressure. The residue was partitioned between EtOAc (50 mL) and 1 M $KHSO_4$ (50 mL); the organic phase was washed with 1 M $KHSO_4$ (2 × 50 mL), 1 M $NaHCO_3$ (2 × 50 mL), and brine (50 mL), then dried over $MgSO_4$. Filtration and solvent removal afforded Boc-L-Tyr(^{t}Bu)-Z-ΔAbu-OMe (**6b**) as a yellow oil (0.48 g, 43%). ^{1}H NMR (400 MHz, DMSO-d_6, δ): 1.25 (9H, s, 3 x CH_3 ^{t}Bu), 1.29 (9H, s, 3 x CH_3 Boc), 1.61 (3H, d *J*=7.2, CH_3 ΔAbu), 2.69-2.75 (1H, m, β-CH_2 Tyr), 2.92-2.97 (1H, m, β-CH_2 Tyr), 3.63 (3H, s, OCH_3), 4.24-4.30 (1H, m, α-C*H* Tyr), 6.53 (1H, q *J*=7.2 Hz, β-C*H* ΔAbu), 6.85 (2H, d *J*=8 Hz, Ar*H* Tyr), 6.94 (1H, d *J*=8.8 Hz, N*H* of Tyr), 7.19 (2H, d *J*=8.4 Hz, Ar*H* Tyr), 9.27 (1H, s, N*H* ΔAbu) ppm. ^{13}C-NMR (100.6 MHz, DMSO-d_6, δ): 13.4 (CH_3 ΔAbu), 28.1 and 28.5 ($OC(CH_3)_3$ ^{t}Bu and Boc), 36.8 (β-CH_2 Tyr), 51.8 (OCH_3), 55.6 (CH), 77.5 and 77.9 ($OC(CH_3)_3$ ^{t}Bu and Boc), 123.3 (CH), 123.7 (CH), 132.4 (CH, β-CH ΔAbu), 153.3 (C), 153.4 (C), 155.2 (C), 164.6 (C=O), 170.8 (C=O), 171.0 (C=O) ppm.

Compound **6c:**

DMAP (0.13 equiv, 0.66 mmol, 0.08 g) and di-tert-butyl dicarbonate (Boc_2O, 1.1 equiv, 5.66 mmol, 1.22 g) were added to a solution of Boc-L-Tyr(^{t}Bu)-L-Ser-OMe (**5c**) (1.0 equiv, 5.14 mmol, 2.17 g) in dry MeCN (10 mL). The reaction mixture was stirred at room temperature overnight, monitoring progress by ^{1}H NMR until complete consumption of the starting material. *N,N,N′,N′*-tetramethylguanidine (TMG, 2% v/v, 0.20 mL) was then added, and stirring at room temperature was continued, again monitoring by ^{1}H NMR until the intermediate was consumed. The solvent was removed under reduced pressure. The residue was partitioned between EtOAc (50 mL) and 1 M $KHSO_4$ (50 mL); the organic layer was washed with 1 M $KHSO_4$ (2 × 50 mL), 1 M $NaHCO_3$ (2 × 50 mL), and brine (50 mL), then dried over $MgSO_4$. Filtration and solvent removal under reduced pressure afforded Boc-L-Tyr(^{t}Bu)-ΔAla-OMe (**6c**) as a yellow oil (1.04 g, 48%). ^{1}H NMR (400 MHz, DMSO-d_6,δ): 1.24 (9H, s, 3 x CH_3), 1.29 (9H, s, 3 x CH_3), 2.65-2.73 (1H, m, β-CH_2 Tyr), 2.87-2.97 (1H, m, β-CH_2 Tyr), 3.75 (3H, s, OCH_3), 4.29-4.34 (1H, m, α-C*H* Tyr), 5.71 (1H, s, β-CH_2 ΔAla), 6.25 (1H, s, β-CH_2 ΔAla), 6.85 (2H, d *J*=8.4, Ar*H*), 7.10-7.21 (3H, m, Ar*H* and N*H* Tyr), 9.23 (1H, s, N*H* ΔAla) ppm. ^{13}C-NMR (100.6 MHz, DMSO-d_6, δ): 28.0 and 28.5

(OC(CH_3)$_3$ tBu and Boc), 36.1 (CH_2), 52.7 (OCH_3), 56.3 (CH), 77.5 and 78.3 (OC(CH_3)$_3$ Boc and ^{t}Bu), 109.0 (CH_2, β-CH_2 ΔAla), 123.3 (CH), 129.7 (CH), 132.1 (C), 155.1 (C), 163.6 (C=O), 171.5 (C=O) ppm.

Compound **8a:**

Boc-L-Tyr(^{t}Bu)-Z-ΔPhe-OMe (**6a**) was dissolved in trifluoroacetic acid (TFA) and stirred at room temperature for 1 hour. Removal of TFA under reduced pressure afforded H-L-Tyr-Z-ΔPhe-OMe·TFA (**7a**) as a brown oil (1.10 g, 2.44 mmol). This residue was dissolved in MeCN (20 mL) and the solution cooled to 0 °C. HBTU (1.2 equiv, 2.93 mmol, 1.11 g) was added, and after 2 minutes, Boc-L-Tyr(^{t}Bu)-OH (1.0 equiv, 2.44 mmol, 0.82 g) and triethylamine (3.0 equiv, 7.32 mmol, 1.02 mL) were added sequentially. The reaction mixture was stirred at room temperature overnight. After completion, the solvent was removed under reduced pressure. The residue was partitioned between EtOAc (50 mL) and 1 M $KHSO_4$ (50 mL). The organic layer was washed with 1 M $KHSO_4$ (2 × 50 mL), 1 M $NaHCO_3$ (2 × 50 mL), and brine (50 mL), then dried over $MgSO_4$. Filtration and solvent removal under reduced pressure afforded Boc-L-Tyr(^{t}Bu)-Tyr-Z-ΔPhe-OMe (**8a**) as a white solid (0.86 g, 53%). ^{1}H NMR (400 MHz, DMSO-d_6, δ): 1.33 (9H, s, 3 x CH_3 ^{t}Bu), 1.34 (9H, s, 3 x CH_3 Boc), 2.55-2.61 (1H, m, β-CH_2 Tyr), 2.71-2.89 (2H, m, β-CH_2 Tyr), 2.97-3.02 (1H, m, β-CH_2 Tyr), 3.68 (3H, s, OCH_3), 4.12-4.18 (1H, m, α-C*H* Tyr), 4.64-4.67 (1H, m, α-C*H* Tyr), 6.64-6.67 (2H, m, Ar*H* Tyr), 6.83 (2H, d *J*=12 Hz, Ar*H* Tyr), 6.97-7.11 (4H, m, Ar*H* Tyr), 7.20 (1H, s, β-C*H* ΔPhe), 7.34-7.57 (5H, m, Ar*H* ΔPhe), 8.04 (2H, d *J*=8.4 Hz, N*H* Tyr), 8.17 (2H, d *J*=7.6 Hz, N*H* Tyr), 9.85 (1H, s, N*H* ΔPhe) ppm. ^{13}C-NMR (100.6 MHz, DMSO-d$_6$, δ): 28.0 and 28.5 (C(CH_3)$_3$ ^{t}Bu and Boc), 36.1 and 37.1 (2 x β-CH_2 Tyr), 52.1 (CH_3, OCH_3), 54.1 and 55.6 (2 x α-CH Tyr), 77.5 and 77.9 (*C*(CH_3)$_3$ Boc and ^{t}Bu), 114.91 (CH), 123.2 (CH), 127.4 (C), 128.5 (CH), 129.6 (CH), 130.0 (CH), 130.2 (CH), 131.6 (β-CH ΔPhe), 132.6 (C), 133.1 (C), 153.2 (C), 155.8 (C), 165.3 (C=O), 171.3 (C=O), 171.5 (C=O), 171.8 (C=O) ppm.

Compound **8b:**

Boc-L-Tyr(^{t}Bu)-Z-ΔAbu-OMe (**6b**) was dissolved in trifluoroacetic acid (TFA) and stirred at room temperature for 1 h. Concentration under reduced pressure afforded H-L-Tyr-Z-ΔAbu-OMe·TFA (**7b**) as a brown oil (0.48 g, 1.23 mmol). This material was dissolved in MeCN (25 mL) and the solution cooled to 0 °C. HBTU (1.2 equiv, 1.47 mmol, 0.56 g) was added, and after 2 min Boc-Tyr(^{t}Bu)-OH (1.0 equiv, 1.23 mmol, ~0.415 g) and triethylamine (3.0 equiv, 3.69 mmol, ~0.51 mL) were added sequentially. The reaction mixture was stirred at room temperature overnight. The solvent was removed under reduced pressure, and the residue was partitioned between EtOAc (50 mL) and 1 M $KHSO_4$ (50 mL). The organic layer was washed with 1 M $KHSO_4$ (2 × 50 mL), 1 M $NaHCO_3$ (2 × 50 mL), and brine (50 mL), then dried over $MgSO_4$. Filtration and concentration afforded Boc-L-Tyr(^{t}Bu)-L-Tyr-Z-ΔAbu-OMe (**8b**) as a white solid (0.38 g, 52%). ^{1}H NMR (400 MHz, DMSO-d_6, δ): 1.23 (9H, s, 3 x CH_3 ^{t}Bu), 1.26 (9H, s, 3 x CH_3 Boc), 1.59 (3H, d *J*=7.2 Hz, CH_3 ΔAbu), 2.74-2.93 (4H, m, 2 x β-CH_2 Tyr), 3.62 (3H, s, OCH_3), 3.99-4.10 (1H, m, α-C*H* Tyr), 4.57-4.62 (1H, m, α-C*H* Tyr), 6.51 (1H, q *J*=7.2 Hz, β-C*H* ΔAbu), 6.64 (2H, d *J*=8.4 Hz, Ar*H* Tyr), 6.85 (4H, d *J*=8.8 Hz, Ar*H* Tyr), 7.93 (2H, d *J*=8.4 Hz, N*H* of Tyr), 9.34 (1H, s, N*H* ΔAbu) ppm. ^{13}C-NMR (100.6 MHz, DMSO-d$_6$, δ): 13.3 (CH_3 ΔAbu), 28.0 and 28.5 (OC(CH_3)$_3$ ^{t}Bu and Boc), 35.7 and 37.1 (2 x β-CH_2 Tyr), 51.8 (OCH_3), 53.8 (CH),

55.1 (CH), 77.5 and 77.7 ($OC(CH_3)_3$ ᵗBu and Boc), 114.8 (CH), 114.9 (CH), 120.9 (CH), 123.2 (CH), 123.3 (CH), 123.4 (CH), 129.6 (CH), 129.8 (CH), 133.6 (β-CH ΔAbu), 153.3 (C), 153.7 (C), 155.8 (C), 164.5 (C=O), 170.1 (C=O), 171.0 (C=O), 171.3 (C=O) ppm.

Compound **8c:**

Boc-Tyr (tBu)-Z-ΔAla -OMe (**6c**) was dissolved in TFA and the mixture was left at rt for 1 hour. TFA removal under reduced pressure secured H-Tyr(OH)-Z-ΔAla-OMe.TFA (**7c**) (2.76 mmol, 1,04 g) as a brown oil, which was dissolved in MeCN (20 mL). The mixture was cooled to 0° C. HBTU (1.2 equiv, 3.31 mmol, 1.25 g) was added, and after 2 minutes Boc-Tyr (tBu)-OH (1.0 equiv, 2.76 mmol, 0.93 g) and triethylamine (3.0 equiv, 8.27 mmol, 1.15 mL) were added sequentially to the mixture, which was left stirring at rt overnight. The solvent was removed under reduced pressure. The addition of EtOAc (50 mL) and $KHSO_4$ (1 M, 50 mL) to the residue afforded the separation of two phases. The organic layer was washed with $KHSO_4$ (1 M, 2 x 50 mL), $NaHCO_3$ (1 M, 2 x 50 mL) and brine (50 mL) and then dried with $MgSO_4$. Filtration followed by removal of the solvent under reduced pressure afforded Boc-Tyr(tBu)-Tyr(OH)-Z-ΔAla-OMe (**8c**) (0.86 g, 53%) as a yellow solid. ^{1}H NMR (400 MHz, DMSO-*d*$_6$,δ): 1.29 (9H, s, 3 x C*H*$_3$), 1.34 (9H, s, 3 x C*H*$_3$), 2.72-2.84 (2H, m, β-C*H*$_2$ Tyr), 2.89-2.91 (1H, m, β-C*H*$_2$ Tyr), 2.93-2.94 (1H, m, β-C*H*$_2$ Tyr), 3.73 (3H, s, OC*H*$_3$), 4.08-4.13 (1H, m, *α*-C*H* Tyr), 4.61-4.67 (1H, m, *α*-C*H* Tyr), 5.71 (1H, s, β-C*H*$_2$ ΔAla), 6.17 (1H, s, β-C*H*$_2$ ΔAla), 6.63 (2H, d *J*=8.4 Hz), Ar*H* Tyr), 6.79-6.87 (2H, m, Ar*H* Tyr and N*H* Tyr), 7.03-7.07 (4H, m, ArH Tyr), 8.07 (2H, d *J*=8 Hz, N*H* Tyr), 9.36 (1H, s, N*H* ΔAla) ppm. ^{13}C-NMR (100.6 MHz, DMSO-d$_6$, δ): 28.0 and 28.5 (OC(*C*H$_3$)$_3$ *ᵗ*Bu and Boc), 36.4 (CH_2) 36.9 (CH_2), 52.6 (OCH_3), 54.5 (CH), 55.7 (CH), 77.5 and 78.0 (O*C*(CH$_3$)$_3$ *ᵗ*Bu and Boc), 109.7 (β-CH_2 ΔAla), 114.8 (CH), 123.2 (CH), 127.2 (C), 129.6 (CH), 130.2 (CH), 132.6 (C), 153.2 (C), 155.8 (C), 163.6 (C=O), 170.7 and 171.7 (C=O) ppm.

Compound **10a:**

Boc-L-Tyr(*ᵗ*Bu)-L-Tyr-*Z*-ΔPhe-OMe (**8a**) was dissolved in trifluoroacetic acid (TFA) and stirred at room temperature for 1 hour. Removal of TFA under reduced pressure afforded H-L-Tyr-L-Tyr-*Z*-ΔPhe-OMe·TFA (**9a**) as a brown oil (0.85 g, 1.38 mmol). The crude product was dissolved in MeCN (20 mL) and cooled to 0 °C. HBTU (1.2 equiv, 1.67 mmol, 0.63 g) was added, and after 2 minutes, 2-(naphth-2-yl)acetic acid (1.0 equiv, 1.39 mmol, 0.26 g) and triethylamine (3.0 equiv, 4.17 mmol, 0.58 mL) were added sequentially. The reaction mixture was stirred at room temperature overnight. The solvent was removed under reduced pressure, and the residue was partitioned between EtOAc (50 mL) and 1 M $KHSO_4$ (50 mL). The organic layer was washed with 1 M $KHSO_4$ (2 × 50 mL), 1 M $NaHCO_3$ (2 × 50 mL), and brine (50 mL), then dried over $MgSO_4$. Filtration and solvent removal under reduced pressure yielded Naph-L-Tyr-L-Tyr-Z-ΔPhe-OMe (**10a**) as a white solid (0.66 g, 70%). ^{1}H NMR (400 MHz, DMSO-*d*$_6$, δ): 2.57-2.63 (1H, m, β-C*H*$_2$ Tyr), 2.70-2.76 (1H, m, β-C*H*$_2$ Tyr), 2.87-2.91 (1H, m, β-C*H*$_2$ Tyr), 2.96-3.01 (1H, m, β-C*H*$_2$ Tyr), 3.46-3.48 (1H, m, C*H*$_2$ Naph), 3.68 (3H, s, OC*H*$_3$), 4.48-4.92 (1H, m, *α*-C*H* Tyr), 4.61-4.62 (1H, m, *α*-C*H* Tyr), 6.55 (2H, d *J*=8.4 Hz, Ar*H* Tyr), 6.65 (2H, d *J*=8.4 Hz, Ar*H* Tyr), 6.97 (2H, d *J*=8.4 Hz, Ar*H* Tyr), 7.08 (2H, d *J*=8.4 Hz, Ar*H* Tyr), 7.19 (1H, s, β-C*H* ΔPhe),

7.32-7.83 (12H, m, Ar*H*), 8.20 (1H, d *J*=8.4 Hz, N*H* Tyr), 8.36 (1H, d *J*=7.2 Hz, N*H* Tyr), 9.82 (1H, s, N*H* ΔPhe) ppm. ^{13}C-NMR (100.6 MHz, DMSO-d$_6$, δ): 36.3 and 36.8 (2 x β-CH$_2$ Tyr), 42.2 (CH$_2$ Naph), 52.1 (CH$_3$, OCH$_3$), 53.9 and 54.3 (2 x α-CH Tyr), 114.1 (CH), 114.9 (CH), 125.4 (CH), 125.9 (CH), 127.2 (CH), 127.5 (CH), 127.6 (C), 127.8 (C), 128.5 (CH), 129.3 (CH), 130.0 (CH), 130.1 (CH), 130.2 (CH), 131.4 (β-CH ΔPhe), 131.6 (C), 132.9 (C), 133.2 (C), 133.9 (C), 155.7 and 155.8 (C), 165.3 (C=O), 169.7 (C=O), 171.3 (C=O) and 171.5 (C=O) ppm.

Compound **10b:**

Boc-L-Tyr(^{t}Bu)-L-Tyr(OH)-*Z*-ΔAbu-OMe (**8b**) was dissolved in trifluoroacetic acid (TFA) and stirred at room temperature for 1 h. Removal of TFA under reduced pressure afforded H-L-Tyr(^{t}Bu)-L-Tyr(OH)-*Z*-ΔAbu-OMe (**9b**) as a brown oil (0.38 g, 0.63 mmol). The residue was dissolved in MeCN (25 mL) and cooled to 0 °C. HBTU (1.2 equiv, 0.76 mmol, 0.29 g) was added, and after 2 min, 2-(naphth-2-yl) acetic acid (1.0 equiv, 0.63 mmol, 0.12 g) and triethylamine (3.0 equiv, 1.89 mmol, 0.26 mL) were added sequentially. The reaction mixture was stirred at room temperature overnight. After completion, the solvent was removed under reduced pressure, and the residue was partitioned between EtOAc (50 mL) and 1 M $KHSO_4$ (50 mL). The organic layer was washed with 1 M $KHSO_4$ (2 × 50 mL), 1 M $NaHCO_3$ (2 × 50 mL), and brine (50 mL), then dried over $MgSO_4$. Filtration and solvent removal under reduced pressure afforded Naph-L-Tyr(^{t}Bu)-L-Tyr(OH)-*Z*-ΔAbu-OMe (**10b**) as a light-yellow solid (0.24 g, 63%). ^{1}H NMR (400 MHz, DMSO-*d*$_6$, δ): 1.58 (3H, d *J*=6.8 Hz, C*H*$_3$ ΔAbu), 2.49-2.96 (4H, m, β-C*H*$_2$ Tyr), 3.46 (2H, s, C*H*$_2$ Naph), 3.62 (3H, s, OC*H*$_3$), 4.45-4.46 (1H, m, α-C*H* Tyr), 4.56-4.58 (1H, m, α-C*H* Tyr), 6.52-6.58 (3H, m, β-C*H* ΔAbu and Ar*H* Tyr), 6.64 (2H, d *J*=8.4 Hz, Ar*H* Tyr), 6.95-6.98 (2H, m, Ar*H* Tyr), 7.05 (2H, d J=8.4 Hz), 7.42-7.48 (3H, m, Ar*H* Naph), 7.73-7.84 (4H, m, Ar*H* Naph), 8.12 (2H, d *J*=8 Hz, N*H* Tyr), 8.17 (2H, d *J*=8.4 Hz, N*H* Tyr), 9.26 (1H, s, N*H* ΔAbu) ppm. ^{13}C-NMR (100.6 MHz, DMSO-d$_6$, δ): 13.3 (CH$_3$ ΔAbu), 36.7 (CH$_2$), 36.9 (CH$_2$), 42.2 (CH$_2$), 54.1 (CH), 114. 8 (CH), 114.9 (CH), 125.4 (CH), 125.9 (CH), 127.2 (CH), 127.4 (CH), 127.5 (CH), 130.1 (CH), 130.2 (CH), 131.7 (C), 132.6 (β-C*H* ΔAbu), 132.9 (C), 133.9 (C), 155.7 (C), 155.9 (C), 164.5 (C=O), 169.8 (C=O), 170.2 (C=O), 171.2 (C=O) ppm.

Compound **10c:**

Boc-L-Tyr(^{t}Bu)-L-Tyr-ΔAla-OMe (**8c**) was dissolved in trifluoroacetic acid (TFA) and stirred at room temperature for 1 h. Concentration under reduced pressure afforded H-L-Tyr-L-Tyr-ΔAla-OMe·TFA (**9c**) as a brown oil (0.86 g, 1.59 mmol). The residue was dissolved in MeCN (20 mL) and cooled to 0 °C. HBTU (1.2 equiv, 1.91 mmol, 0.72 g) was added, and after 2 min 2-(naphth-2-yl)acetic acid (1.0 equiv, 1.59 mmol, 0.30 g) and triethylamine (3.0 equiv, 4.77 mmol, 0.67 mL) were added sequentially. The reaction mixture was stirred at room temperature overnight. The solvent was removed under reduced pressure, and the residue was partitioned between EtOAc (50 mL) and 1 M $KHSO_4$ (50 mL). The organic layer was washed with 1 M $KHSO_4$ (2 × 50 mL), 1 M $NaHCO_3$ (2 × 50 mL), and brine (50 mL), then dried over $MgSO_4$. Filtration and concentration afforded Naph-L-Tyr(^{t}Bu)-L-Tyr-ΔAla-OMe (**10c**) as a light-yellow solid (0.69 g, 73%). ^{1}H NMR (400 MHz, DMSO-*d*$_6$,δ): 2.57-2.77 (2H, m, β-C*H*$_2$ Tyr), 2.84-2.94 (2H, m, β-C*H*$_2$ Tyr), 3.51 (2H, s, C*H*$_2$ Naph), 3.73 (3H, s, OC*H*$_3$), 4.43-4.47 (1H, m, α-C*H* Tyr), 4.59-4.62 (1H, m, α-C*H* Tyr), 5.71 (1H, s, β-C*H*$_2$ ΔAla), 6.18 (1H, s, β-C*H*$_2$ ΔAla), 6.56 (2H, d *J*=8.4

Hz, ArH Tyr), 6.63 (2H, d *J*=8.4 Hz, ArH Tyr), 6.95 (2H, d *J*=8.8 Hz, ArH Tyr), 7.03 (2H, d *J*=8.4 Hz, ArH Tyr), 7.18-7.84 (7H, m, Ar*H* Naph), 8.19 (1H, d *J*=8.4 Hz, N*H* Tyr), 8.26 (1H, d *J*=8 Hz, N*H* Tyr), 9.28 (1H, s, N*H* ΔAla) ppm. ^{13}C-NMR (100.6 MHz, DMSO-d$_6$, δ): 36.1 (CH_2), 36.7 (CH_2), 42.1 (CH_2), 52.6 (OCH_3), 54.0 (CH), 54.7 (CH), 109.5 (CH_2), 114.7 (CH), 114.8 (CH), 115.7 (C), 118.7 (C), 121.7 (C), 124.3 (CH), 125.9 (CH), 127.3 (CH), 127.4 (CH), 127.7 (C), 127.8 (C), 130.0 (CH), 130.1 (CH), 131.6 (C), 132.3 (C), 132.9 (C), 133.9 (C), 155.7 (C), 155.8 (C), 163.3 (C=O), 169.7 (C=O), 170.7 and 171.5 (C=O) ppm.

Compound **11:**

Boc-L-Tyr(^{t}Bu)-L-Tyr-*Z*-ΔPhe-OMe (**8a**) (0.37 g, 0.55 mmol) was dissolved in 1,4-dioxane (10 mL), and 1.0 M NaOH (3.0 equiv, 1.66 mmol, 1.66 mL) was added. The reaction was stirred at room temperature and monitored by TLC until complete consumption of the starting material (≈4.5 h). Distilled water was added, and the solvent was removed under reduced pressure. The residue was acidified with HCl and left at 4 °C overnight. The precipitate was collected by vacuum filtration to afford Boc-L-Tyr(^{t}Bu)-L-Tyr-*Z*-ΔPhe-OH (**11**) as a light-brown solid (0.28 g, 78%). ^{1}H NMR (400 MHz, DMSO-*d$_6$*, δ): 1.26 (9H, s, 3 x CH_3 ^{t}Bu), 1.29 (9H, s, 3 x CH_3 Boc), 2.72-2.83 (2H, m, β-CH_2 Tyr), 2.97-3.02 (2H, m, β-CH_2 Tyr), 4.13-4.18 (1H, m, α-C*H* Tyr), 4.63-4.67 (1H, m, α-C*H* Tyr), 6.64 (2H, d *J*=8.4 Hz, Ar*H* Tyr), 6.81 (2H, m, Ar*H* Tyr), 7.04-7.10 (4H, m, Ar*H* Tyr), 7.25 (1H, s, β-C*H* ΔPhe), 7.33-7.56 (5H, m, Ar*H* ΔPhe), 8.00 (2H, d *J*=8 Hz, N*H* Tyr), 8.17 (2H, d *J*=7.6 Hz, N*H* Tyr), 9.67 (1H, s, N*H* ΔPhe), 12.63 (1H, br s, O*H*) ppm.

Compound **12:**

H-L-Tyr-L-Tyr-*Z*-ΔPhe-OMe·TFA (**11**) (0.57 g, 0.92 mmol) was dissolved in MeCN (25 mL) and cooled to 0 °C. HBTU (1.2 equiv, 1.11 mmol, 0.42 g) was added, and after 2 minutes, Boc-L-Tyr(^{t}Bu)-OH (1.0 equiv, 0.92 mmol, 0.31 g) and triethylamine (3.0 equiv, 2.77 mmol, 0.38 mL) were added sequentially. The reaction mixture was stirred at room temperature overnight. The solvent was removed under reduced pressure, and the residue was partitioned between EtOAc (50 mL) and 1 M $KHSO_4$ (50 mL). The organic layer was washed with 1 M $KHSO_4$ (2 × 50 mL), 1 M $NaHCO_3$ (2 × 50 mL), and brine (50 mL), then dried over $MgSO_4$. Filtration and solvent removal under reduced pressure afforded Boc-L-Tyr(^{t}Bu)-L-Tyr-L-Tyr-*Z*-ΔPhe-OMe (**12**) as a yellow solid (0.51 g, 67%). ^{1}H NMR (400 MHz, DMSO-*d$_6$*, δ): 1.28 (9H, s, 3 x CH_3 ^{t}Bu), 1.34 (9H, s, 3 x CH_3 Boc), 2.70-2.79 (1H, m, β-CH_2 Tyr), 2.88-3.01 (1H, m, β-CH_2 Tyr), 3.63 (3H, s, OCH_3), 4.04-4.06 (1H, m, α-C*H* Tyr), 4.49-4.64 (2H, m, 2 x α-C*H* Tyr), 6.56-7.56 (18H, m, Ar*H* and β-C*H* Tyr), 7.81 (1H, d *J*=8.4 Hz, N*H* Tyr), 8.03 (1H, d *J*=8.4 Hz, N*H* Tyr), 8.29 (1H, d *J*=8.4 Hz, N*H* Tyr), 9.82 (1H, s, N*H* ΔPhe) ppm. ^{13}C-NMR (100.6 MHz, DMSO-d$_6$, δ): 28.1 and 28.5 (OC(CH_3)$_3$ ^{t}Bu and Boc), 36.4 (CH_2), 36.9 (CH_2), 37.2 (CH_2), 52.1 (OCH_3), 53.7 (CH), 54.3 (CH,), 57.3 (CH), 77.5 and 77.9 (OC(CH_3)$_3$ Boc and ^{t}Bu), 114.8 (CH), 114.9 (CH), 124.6 (C), 125.9 (C), 126.0 (C), 127.3 (C), 128.5 (CH), 129.2 (CH), 129.7 (CH), 130.1 (CH), 130.3 (CH), 132. 8 (CH, β-CH ΔPhe), 133.2 (C), 155.7 (C), 155.9 (C), 165.3 (C=O), 168.0 (C=O), 171.2(C=O), 171.4 (C=O) ppm.

Compound **13:**

Boc-L-Tyr(^{t}Bu)-L-Tyr-L-Tyr-*Z*-ΔPhe-OMe (**12**) (0.50 g, 0.61 mmol) was dissolved in 1,4-dioxane (10 mL), and 1 M NaOH (3.0 equiv, 1.83 mmol, 1.83 mL) was added. The reaction was stirred at room temperature and monitored by TLC until complete consumption of the starting material (≈ 5 h). Distilled water was then added, and the solvent was removed under reduced pressure. The residue was acidified with HCl and left at 4 °C overnight. The resulting precipitate was collected by vacuum filtration to afford Boc-L-Tyr(^{t}Bu)-L-Tyr-L-Tyr-*Z*-ΔPhe-OH (**13**) as a light-brown solid (0.43 g, 88 %). ^{1}H NMR (400 MHz, DMSO-d_6, δ): 1.23 (9H, s, 3 x CH_3 ^{t}Bu), 1.25 (9H, s, 3 x CH_3 Boc), 2.73-2.89 (3H, m, β-CH_2 Tyr), 2.99-3.03 (3H, m, β-CH_2 Tyr), 4.03-4.07 (1H, m, α-C*H* Tyr), 4.47-4.94 (1H, m, α-C*H* Tyr), 4.62-4.64 (1H, m, α-C*H* Tyr), 6.56-7.09 (12H, m, Ar*H* Tyr), 7.23 (β-C*H* ΔPhe), 7.32-7.55 (ArH ΔPhe), 7.75 (1H, d *J*=8.8 Hz, N*H* Tyr), 8.23 (1H, d *J*=7.6 Hz, N*H* Tyr), 9.64 (1H, s, N*H* ΔPhe), 12.04 (1H, br s, O*H*) ppm.
